# Reactive sulfur species as emerging immunomodulators: mechanistic insights and therapeutic prospects

**DOI:** 10.3389/fimmu.2026.1736794

**Published:** 2026-01-28

**Authors:** Youbang Chen, Ruiying Ji, Yixing Wu, Xiang Li, Hui Zhang, Chun-tao Yang

**Affiliations:** 1 Guangzhou Municipal and Guangdong Provincial Key Laboratory of Protein Modification and Disease, School of Basic Medical Sciences, Guangzhou Medical University, Guangzhou, China; 2 Jinzhuan Biotechnology (China) Research Institute, Guangzhou, China

**Keywords:** adaptive immunity, immunoregulation, innate immunity, metabolic disease, neutrophil NETosis, reactive sulfur species

## Abstract

Inflammation is a vital component of host defense and tissue repair, but its dysregulation contributes to chronic metabolic and immune-mediated diseases. In recent years, reactive sulfur species (RSS) have emerged as crucial regulators of immune homeostasis. Unlike reactive oxygen and nitrogen species, RSS dynamically regulates cellular signaling networks through reversible protein persulfidation. Rather than exerting uniformly pro- or anti-inflammatory actions, RSS display context-dependent, bidirectional effects that fine-tune immune responses according to the cellular redox state, metabolic and inflammatory conditions. This review integrates current advances in understanding how RSS mediate immune regulation across both innate and adaptive systems. We discuss how RSS shape macrophage polarization, modulate neutrophil activation and NETosis, influence dendritic cell differentiation, and control T and B cell function. We further examine translational efforts employing diverse RSS donors, including H_2_S-releasing compounds, persulfide and polysulfide donors, and engineered biomaterial delivery systems, to achieve targeted immune modulation. Finally, we highlight key challenges, such as context specificity, donor controllability, and redox balance, that must be resolved to realize the therapeutic potential of RSS.

## 1 Introduction

### 1.1 Immune-metabolic dysregulation in metabolic diseases: an unmet therapeutic need

Immune dysregulation underlies a broad range of human diseases, including metabolic disorders such as type 2 diabetes mellitus (T2DM), obesity, and metabolic dysfunction-associated steatohepatitis (MASH) (1–4); autoimmune conditions exemplified by multiple sclerosis (MS), systemic lupus erythematosus (SLE), and inflammatory bowel disease (IBD) (5–7); and even cancer (8, 9). In metabolic diseases, chronic low-grade inflammation often drives systemic complications, linking immune dysfunction to metabolic stress and multi-organ injury (10–12). In autoimmune disorders, aberrant immune cell activation causes tissue damage (13). In cancer, immunosuppressive microenvironments facilitates immune evasion through multiple mechanisms, including regulatory T cell (Treg) expansion, myeloid-derived suppressor cell (MDSC) accumulation, and CD8^+^ T cell exhaustion (14–17). Current therapies focus on single pathways but do not correct the fundamental redox and metabolic disturbances that regulate immune responses. Therefore, finding natural mechanisms that connect cellular metabolism with immune function is an important unmet need.

Reactive sulfur species (RSS), emerging as a distinct redox system beyond reactive oxygen species (ROS) and reactive nitrogen species (RNS), exhibit unique immunomodulatory properties (18–20). Recent conceptual advances in redox immunology emphasize that immune responses are regulated through integrated networks of reactive species. In particular, the “reactive species interactome,” consisting of ROS, RNS and RSS, provides spatially and temporally organized redox signals that coordinate innate immune activation, metabolic remodeling, and inflammatory resolution (21). Positioning RSS within this broader redox signaling paradigm helps interpret sulfur-dependent immune mechanisms.

The RSS family, comprising hydrogen sulfide (H_2_S), persulfides, polysulfides, and related sulfur-based modifications, exerts immunoregulatory functions primarily through protein persulfidation (S-Sulfhydration), a post-translational modification that alters protein conformation, activity, and interactions (22–26). Crucially, patients with metabolic diseases often display reduced endogenous H_2_S generation and downregulated expression of RSS-producing enzymes (cystathionine β-synthase, CBS; cystathionine γ-lyase, CSE), correlating with inflammatory complications and tissue damage (27–29). This deficiency-disease association positions RSS as a promising therapeutic target at the immune-metabolic interface.

### Scope and organization of this review

This review systematically examines RSS immunoregulatory mechanisms across both innate and adaptive immunity, emphasizing their pathophysiological relevance to metabolic diseases. We first introduce the chemical and biological foundations of RSS (Section 2), including their enzymatic biosynthesis, dynamic interconversion networks, and signaling mechanisms. We then dissect RSS roles in innate immune cells (Section 3), focusing on macrophages, neutrophils and dendritic cells, with emphasis on therapeutic implications in diabetic wounds, obesity-related inflammation and autoimmune disorders. Section 4 explores RSS functions in adaptive immunity, including T cell activation, regulatory T cell differentiation, CD8^+^ T cell ferroptosis resistance, and RSS-mediated CD4^+^ T cell regulation, alongside preliminary evidence in B cell-mediated humoral immunity. Throughout each section, we integrate disease model validation and translational prospects, from molecular donors to innovative biomaterial delivery systems. Finally, Section 5 identifies critical knowledge gaps (context-dependent molecular switches, donor specificity, dose-response relationships) and outlines future research directions bridging basic immunology with clinical translation.

**Figure 1:**
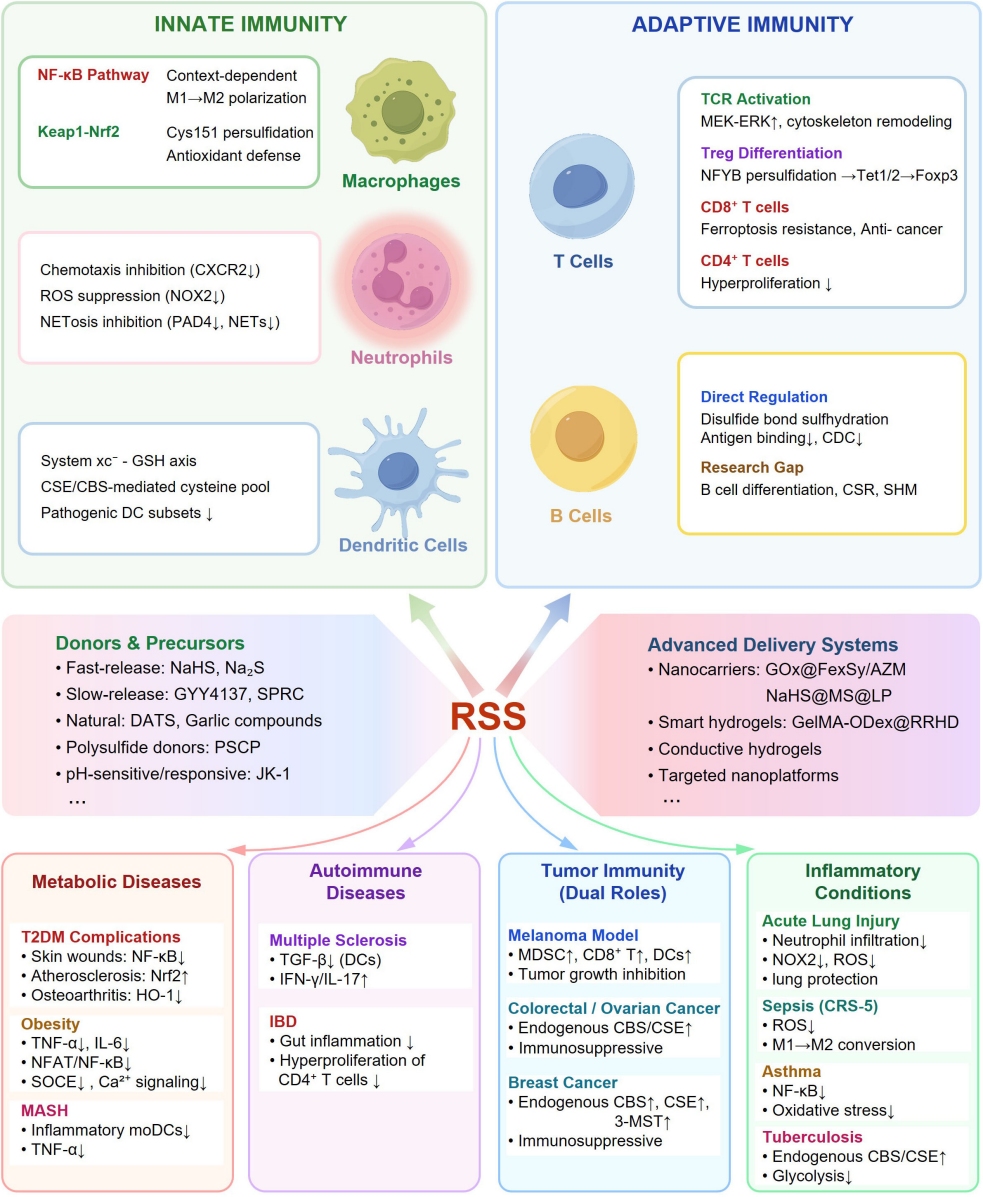
Comprehensive overview of RSS-mediated immune regulation and therapeutic applications. RSS function as central immunomodulators integrating innate and adaptive immunity through hierarchical mechanisms. Innate immunity(green): RSS regulate macrophage polarization via NF-κB and Nrf2 pathways, suppress neutrophil NETosis through ROS-MAPK-PAD4 axis inhibition, and modulate DC function via cysteine-GSH metabolic control. Adaptive immunity (blue): RSS enhance T cell activation, stabilize Treg differentiation through NFYB-Tet1/2 epigenetics, protect CD8^+^ T cells from ferroptosis, and regulate B cell antibody structure via disulfide persulfidation. RSS donors span fast-release (NaHS), slow-release (GYY4137), natural (DATS), polysulfide (PSCP), and pH-sensitive (JK-1) compounds. Advanced delivery systems include responsive nanocarriers, smart hydrogels, and targeted platforms. Therapeutic applications encompass metabolic diseases (T2DM, obesity, MASH), autoimmune disorders (MS, IBD), tumor immunity (melanoma, colorectal/ovarian/breast cancers), and inflammation (ALI, sepsis, asthma, Tuberculosis). This framework positions RSS as precision immunomodulators bridging redox metabolism and immune programming.

Through this framework, we aim to provide both mechanistic insights into RSS-mediated immune regulation and a strategy for developing RSS-based precision therapeutics for metabolic and immune-related diseases (**Figure 1**).

## 2 Chemical and biological basis of RSS

### 2.1 Chemical diversity and dynamic transformation network of RSS

RSS refers to a class of chemically active sulfur-containing molecules and functional groups. The biological uniqueness of RSS originates from sulfur’s rich redox chemistry: sulfur atoms can transition between multiple oxidation states ranging from -2 to +6, endowing RSS with excellent redox reactivity and signal transduction capacity (30, 31). The RSS family primarily includes hydrogen sulfide (H_2_S), persulfides (RSSH), polysulfides (RSS_n_H and H_2_S_n_, n≥2), and sulfur oxoacids including sulfite (SO_3_^2−^) and thiosulfate (S_2_O_3_^2−^) (32, 33). These RSS undergo dynamic interconversion via redox reactions, forming an interconnected sulfur metabolic network (34, 35).

H_2_S, as the core precursor molecule, exists primarily as HS^−^ ions (pKa ≈ 7.0) under physiological conditions and serves as the biochemical hub for generating other RSS. Persulfides, formed through H_2_S reaction with protein cysteine residues or disulfide bonds, possess stronger nucleophilicity and reducing capacity than thiols (RSH), with significantly lower pKa values (≈4–6 vs. ≈8-9), making them more readily ionized and reactive at physiological pH (18, 35). Organic polysulfides (RSSnR or RSSnH, n≥2) and inorganic polysulfides (H_2_SSn, n≥2) are formed through sulfur-sulfur bond linkages and serve dual roles (1): storage and transport forms of reactive sulfur species, and (2) independent signaling molecules. Notably, inorganic polysulfides exhibit electrophilic character distinct from H_2_S’s nucleophilicity, enabling direct protein thiol modification without requiring intermediate oxidation steps (36, 37). This unique property positions polysulfides as bridging molecules between RSS and ROS/RNS signaling networks, with context-dependent immunomodulatory functions. Sulfane sulfur (S^0^) in persulfides and polysulfides, can donate zero-valent sulfur under enzymatic or non-enzymatic conditions, promoting RSS interconversion and mediating protein persulfidation (38, 39).

Beyond the H_2_S-persulfide-polysulfide system, sulfur oxoacids play immunoregulatory roles. Sulfite (SO_3_^2-^), generated through sulfur amino acid catabolism or as an intermediate in mitochondrial H_2_S oxidation (Section 2.2.2), serves as both a signaling molecule and antioxidant (40). In immune cells, sulfite modulates neutrophil oxidative burst and macrophage cytokine production through mechanisms different from H_2_S-mediated persulfidation (41). Thiosulfate, the terminal product of the SQOR-ETHE1-TST pathway, functions as a stable sulfur reservoir that can be reduced back to H_2_S under specific conditions, serving as an endogenous sulfur buffer system (42, 43).

These interconversions among RSS are precisely regulated by cellular redox status, and enzymatic reactions, collectively constituting a dynamic equilibrium network of intracellular sulfur metabolism. This metabolic flexibility enables immune cells to fine-tune their redox status in response to inflammatory cues, with different RSS species mediating distinct signaling outcomes based on their electrophilic/nucleophilic properties and target protein selectivity.

### 2.2 Metabolism and signaling of endogenous RSS

#### 2.2.1 H_2_S generation: tissue-specific enzyme distribution and regulatory properties

Intracellular H_2_S generation primarily depends on three enzymes (44): CBS (liver, kidney, brain) can be activated by SAM and translocates to mitochondria under stress, coupling RSS signaling to energy metabolism (45, 46). CSE (liver, kidney, vasculature, immune cells) exhibits Ca^2+^-responsive regulation via calmodulin, enabling rapid H_2_S generation during immune activation (47–49). 3-MST shows unique dual cytoplasmic-mitochondrial localization, serving as the primary mitochondrial H_2_S source (50, 51). These spatially and temporally regulated enzymes create RSS generation nodes across immune cell types, while cysteine dioxygenase 1 (CDO1) indirectly regulates RSS levels by controlling substrate availability (52, 53).

#### 2.2.2 Mitochondrial H_2_S oxidation: closing the metabolic loop

H_2_S oxidative metabolism represents a critical regulatory axis controlling cellular RSS levels. In mitochondria, H_2_S undergoes sequential enzymatic oxidation through sulfide quinone oxidoreductase (SQOR), persulfide dioxygenase (ETHE1), thiosulfate sulfurtransferase (TST) and sulfite oxidase (SUOX), ultimately converting to thiosulfate and sulfate while feeding electrons into the respiratory chain (54, 55).

SQOR catalyzes the initial step, oxidizing H_2_S and transferring electrons to coenzyme Q, directly coupling sulfide oxidation to ATP production (54). Critically, SQOR uses glutathione (GSH) as the primary sulfur acceptor to form glutathione persulfide (GSSH). This GSSH intermediate serve dual roles: (1) as a substrate for downstream catabolic enzymes, and (2) as a mobile persulfide donor that diffuses from mitochondria to participate in cytosolic RSS signaling. Thus, SQOR functions both as a catabolic enzyme controlling H_2_S levels and as a generator of signaling-competent persulfide intermediates. ETHE1 subsequently oxidizes persulfides (primarily GSSH) to sulfite. TST then catalyzes the reaction between another GSSH molecule and SO_3_^2-^ to produce thiosulfate as a stable product. Sulfite can also be further oxidized by SUOX to sulfate (SO_4_^2-^) (55). The genetic importance of this pathway is underscored by ethylmalonic encephalopathy (ETHE1 mutations), where toxic H_2_S accumulation impairs mitochondrial function and immune responses, such as chronic inflammation and neutrophil dysfunction (56).

Collectively, the coordinated action of biosynthetic enzymes (CBS, CSE, 3-MST, CDO1) and catabolic enzymes (SQOR, ETHE1, TST, SUOX) constitutes an integrated RSS metabolic network. The spatiotemporal regulation of their expression, activity, and subcellular localization determines RSS levels and speciation across different immune cell types and physiological-pathological states, providing multiple therapeutic intervention points for immune-metabolic diseases.

### 2.3 Protein persulfidation: the molecular basis of RSS signal transduction

The core mechanism by which RSS exerts biological function is achieved via persulfidation of protein cysteine residues. Persulfidation refers to the covalent modification process in which the thiol group (-SH) of protein cysteine side chains is converted to persulfide (-SSH), a modification that significantly alters the chemical reactivity, structural stability, and functional activity of target proteins (38, 57).

Persulfidation modification can occur through multiple pathways: H_2_S can react with disulfide bonds in protein cysteine residues or with oxidatively modified cysteine (such as sulfenic acid -SOH) to generate persulfides; persulfides can also be directly introduced through trans-persulfidation reactions of polysulfides. This modification exhibits high redox reversibility and can be reduced by glutathione or the thioredoxin system, making it a dynamic regulatory node in cellular redox signal transduction (35, 38).

In immune cells, persulfidation has been demonstrated to regulate key signaling pathways. Kelch-like ECH-associated protein 1 (Keap1) protein contains multiple highly reactive cysteine residues (such as Cys151, Cys273, Cys288), and its persulfidation modification disrupts the binding between Keap1and Nuclear factor erythroid 2-related factor 2 (Nrf2), leading to Nrf2 stabilization and nuclear translocation to initiate antioxidant gene expression (58, 59). Additionally, the RelA/p65 subunit of the nuclear factor-κB (NF-κB) pathway has been reported to be regulated by persulfidation, whereas such modification has not yet been clearly demonstrated for the p50 subunit or upstream IKK components. Persulfidation of p65 at cysteine-38 promotes its binding to the coactivator ribosomal protein S3 (RPS3), thereby enhancing NF-κB-dependent transcription of anti-apoptotic genes and contributing to cellular protection against cytokine-induced cell death (60). The active site of protein tyrosine phosphatases (PTPs) contains low pKa cysteine residues, typically in the highly reactive thiolate anion form, making them highly sensitive to RSS. Studies have shown that H_2_S can inhibit PTP1B phosphatase activity by inducing persulfidation (61). Notably, persulfidation modification also serves as a critical link in signal transmission between apoptotic cells and the immune system. H_2_S inhibits abnormal differentiation of Th17 cells by persulfidating cysteine 38 of selenoprotein F, thereby maintaining immune homeostasis and preventing autoimmune diseases (62).

### 2.4 Interactions of RSS with ROS/RNS

RSS do not operate independently but constitute an interconnected redox regulatory network with ROS and RNS. While all three families can modulate cellular redox status and protein function, they exhibit distinct chemical reactivities, biological targets, and signaling mechanisms that determine immune cell states (21, 63).

Direct chemical interactions between RSS and ROS/RNS generate novel bioactive species and modulate redox balance. H_2_S directly scavenges superoxide anion (O_2_•^−^) and hydrogen peroxide (H_2_O_2_), functioning as an antioxidant (64). This ROS-buffering activity is particularly relevant in immune cells, where H_2_S maintains oxidative “setpoints” necessary for proper immune function—limiting pathological ROS accumulation without impairing physiological ROS signals required for antimicrobial responses (65). Beyond direct scavenging, RSS, ROS, and RNS competitively modify protein cysteine residues through distinct chemical mechanisms. Persulfidation exhibits strong nucleophilic character, enabling reduction of oxidized cysteine species and protection against irreversible over-oxidation (66). This reducing capacity allows RSS to reverse certain ROS-mediated modifications, effectively functioning as “antioxidant signals” (67).

RSS further enhance cellular antioxidant capacity by upregulating antioxidant enzymes (superoxide dismutase, catalase, glutathione peroxidase) through Nrf2 pathway activation (68–70). This dual mechanism, combining direct ROS scavenging with transcriptional antioxidant boost, creates robust redox homeostasis. In immune cells, the relative abundance and spatial distribution of RSS, ROS, and RNS determine functional outcomes: activated neutrophils and M1 macrophages exhibit high ROS/low RSS ratios favoring oxidative signaling, while M2 macrophages and regulatory T cells show elevated H_2_S/reduced ROS patterns that support anti-inflammatory programs (Sections 3.1, 4.1).

**Figure 2:**
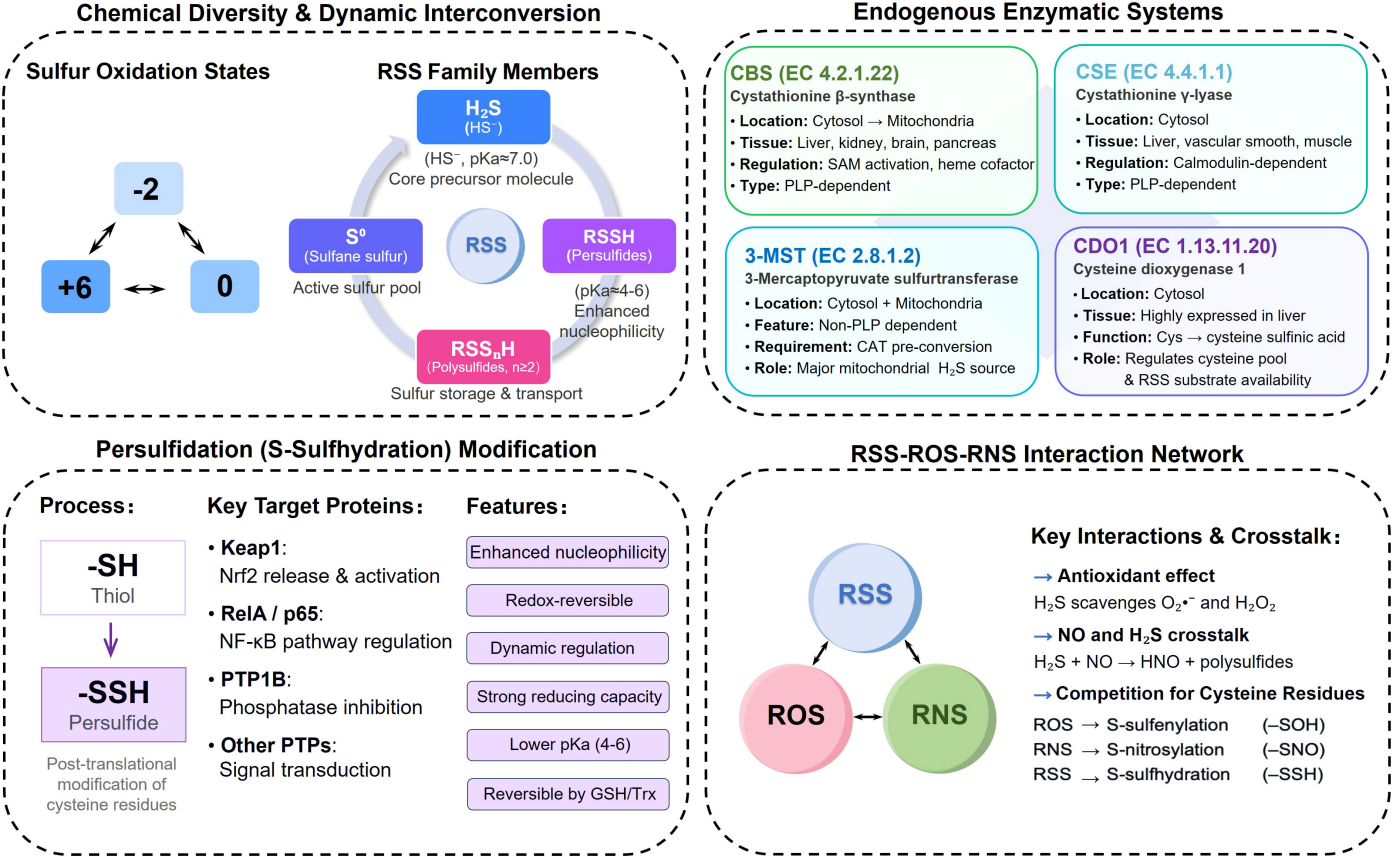
Chemical and biological foundations of RSS: (Top left) Chemical diversity of RSS showing sulfur’s versatile oxidation states (-2 to +6) enabling diverse RSS species, including H_2_S, persulfides (RSSH), polysulfides (RSS_n_H), and sulfane sulfur (S^0^), which constitute a dynamic interconversion network. (Top right) Endogenous enzymatic systems including CBS, CSE, and 3-MST catalyzing H_2_S production with distinct tissue distributions and subcellular localizations, while CDO1 regulates substrate availability. (Bottom left) Persulfidation modification depicting RSS conversion of protein cysteine thiols (-SH) to persulfides (-SSH), modulating key signaling proteins. (Bottom right) Through redox crosstalk, RSS interact with ROS or RNS, scavenging O_2_•^−^/H_2_O_2_, forming polysulfides/HNO, and competitively modifying cysteine residues (-SOH, -SNO, -SSH), to maintain cellular redox homeostasis.

In summary, RSS interact with ROS and RNS through: (1) direct chemical reactions generating bioactive products (polysulfides, HNO); (2) competitive modification of protein cysteine residues and (3) transcriptional antioxidant regulation. These multilevel interactions create a complex redox interactome and determine immune cell functional states. Understanding this integration is essential for developing RSS-based therapeutics that achieve targeted immune modulation without disrupting physiological redox signaling (**Figure 2**).

## 3 RSS and innate immunity

### 3.1 Monocyte-macrophage plasticity: redox-dependent polarization

#### 3.1.1 Context-dependent NF-κB modulation: from acute inflammation to diabetic complications

NF-κB is a master regulator of macrophage inflammatory responses and M1 polarization (71–73). RSS modulates this pathway through three mechanistic tiers that collectively determine context-dependent outcomes: transcriptional regulation, post-translational modification, and metabolic crosstalk. Understanding these multilevel interactions provides mechanistic rationale for RSS-based interventions in diseases featuring NF-κB hyperactivation coupled with endogenous H_2_S deficiency (74).

##### 3.1.1.1 Tier 1: Transcriptional regulation via the HO-1/CO axis and sulfur metabolic feedback

Early mechanistic studies established that H_2_S limits acute inflammation by inducing oxygenase-1 (HO-1). Fast- and slow-release donors (NaHS, GYY4137) upregulate HO-1 via ERK pathway, generating carbon monoxide (CO) that suppresses NF-κB nuclear translocation and downstream iNOS/NO production (75, 76). Beyond this HO-1 pathway, multi-omics analyses reveal that LPS-stimulated macrophages uptake cystine (via SLC7A11/xCT) primarily to generate reactive persulfides and polysulfides (such as CysSSH and GSSH), not just glutathione, thereby suppressing inflammatory responses through a negative feedback loop (77, 78). Persulfidomics mapping shows that the xCT-CSE axis drives widespread protein persulfidation (engaging ∼2% of total thiols and modifying >800 proteins), functioning as a protective brake against oxidative-inflammatory stress. Critically, SQOR acts as a negative regulator of S-persulfidation. Its inhibition elevates cellular persulfide levels, which is associated with increased macrophage resistance to oxidative stress and protection against inflammatory cell death (79). This expanded mechanistic framework reframes H_2_S action from a single HO-1 pathway to a multi-layered sulfur metabolic network that couples persulfide generation with cysteine redox homeostasis.

##### 3.1.1.2 Tier 2: Post-translational modification and metabolic integration

Beyond transcriptional control, H_2_S directly modifies NF-κB through site-specific persulfidation. In synovial macrophages, GYY4137 induces p65 persulfidation (particularly at Cys38), preserving mitochondrial membrane potential and suppressing pyroptosis-associated cytokine release (IL-1β, IL-18) (80–82). This coupling of p65 persulfidation with mitochondrial homeostasis reveals a non-canonical mechanism whereby RSS achieve coordinated redox regulation and anti-inflammatory signaling.

H_2_S further integrates with metabolic pathways to suppress NF-κB. In high-glucose-treated monocytes, CSE-dependent H_2_S generation activates the PI3K/PIP3 axis, upregulating AMP-activated protein kinase (AMPK) phosphorylation and PPARγ expression to reduce ROS generation and inhibit NF-κB phosphorylation (83). In obesity and diabetes, where free fatty acid overload triggers TLR4-NF-κB-NLRP3 inflammasome activation, H_2_S donors inhibit this cascade at multiple nodes: blocking NF-κB activation and downregulating inflammatory cytokine levels (84, 85).

##### 3.1.1.3 Tier 3: Bidirectional regulation—suppression versus stimulation

A critical unresolved question is how identical H_2_S donors produce opposite NF-κB effects depending on inflammatory context. Under homeostatic conditions lacking inflammatory cues, H_2_S exhibits immunostimulatory effects. Korean bamboo salt activates NF-κB in RAW264.7 macrophages to promote TNF-α secretion, with chronic oral administration elevating serum IFN-γ, IL-2, and TNF-α to enhance immune function (86). This bidirectional pattern—suppression during hyperinflammation versus enhancement during quiescence—positions H_2_S as a homeostatic regulator, yet the molecular switches governing this duality remain undefined. Potential mechanisms include differential NF-κB subunit persulfidation under varying redox states, metabolic reprogramming affecting downstream pathway availability, or concentration-dependent receptor engagement.

##### 3.1.1.4 Disease validation and therapeutic translation

The mechanistic insights above converge on a common pathological axis: RSS deficiency → chronic NF-κB hyperactivation → tissue damage. In diabetic wounds, CSE deficiency correlates with sustained p65 phosphorylation, enhanced M1 infiltration, elevated pro-inflammatory cytokines, and activated necroptosis. NaHS supplementation reverses these abnormalities by normalizing p65 phosphorylation, promoting M2 polarization, and inhibiting necroptotic signaling (74, 87). However, traditional donors suffer from rapid release kinetics. Mesoporous silica-liposome systems (NaHS@MS@LP) achieve sustained H_2_S release for more effective wound healing (88), exemplifying evolution toward precision delivery platforms.

Despite progress, critical questions remain: How do different RSS species selectively target NF-κB components under varying inflammatory contexts? Can endogenous RSS enzyme profiles predict therapeutic responses? Addressing these gaps will enable rational design of RSS therapies that restore physiological immune homeostasis.

#### 3.1.2 Activating the Keap1–Nrf2 axis: complementary antioxidant defense

While NF-κB governs transcriptional inflammatory programs (Section 3.1.1), the Keap1-Nrf2 pathway provides a complementary antioxidant defense mechanism. Nrf2 serves as the master regulator of cellular antioxidant responses. Under physiological conditions, Keap1 sequesters Nrf2 in the cytoplasm for continuous proteasomal degradation. RSS activate this pathway through site-specific Keap1 cysteine modification, mimicking oxidative stress signals to initiate protective responses.

##### 3.1.2.1 Molecular mechanism: site-specific Cys151 persulfidation

In diabetic atherosclerosis models, the slow-release donor GYY4137 attenuates aortic plaque formation, oxidative stress, and vascular inflammation in STZ-induced LDLr^−^/^−^ mice, such effects are completely abolished in Nrf2^−^/^−^ mice (89). This indicates that Nrf2 is essential for H_2_S-mediated atheroprotection. Mechanistically, H_2_S induces S-persulfidation of Keap1 at Cys151, disrupting Keap1-Nrf2 binding to promote nuclear translocation and HO-1 expression. Mutation of this residue abolishes GYY4137’s effects (88), suggesting Cys151 as the critical persulfidation site. Sulforaphane (SFN), another Nrf2 activator, operates through a different mechanism, electrophilic modification of multiple Keap1 cysteines, rather than the site-specific persulfidation induced by RSS in mouse peritoneal macrophages (90).

##### 3.1.2.2 Therapeutic convergence on HO-1 despite distinct mechanisms

Despite mechanistic differences, H_2_S and sulforaphane converge on HO-1 as a critical effector. SFN significantly reduces pro-inflammatory mediators in LPS-stimulated macrophages while upregulating HO-1 in an Nrf2-dependent manner (90). In diabetic wound models, topical SFN accelerates healing through dual Nrf2-dependent mechanisms: enhancing macrophage efferocytosis (via HO-1-mediated MERTK upregulation) and promoting M2 polarization (91). This convergence underscores HO-1 as a nodal point integrating RSS-NF-κB and RSS-Nrf2 pathways (recall Section 3.1.1 where HO-1-derived CO suppresses NF-κB).

##### 3.1.2.3 Upregulation of mitochondrial antioxidant enzymes

While HO-1 remains a primary effector of the Keap1-Nrf2 axis, our recent work demonstrates that RSS-mediated Nrf2 activation also regulates other critical protective enzymes. Specifically, we identified that the novel polysulfide donor PSCP preserves mitochondrial function in diabetic retinopathy by upregulating isocitrate dehydrogenase (NADP(+)) 2 (IDH2) and microsomal glutathione S-transferase 1 (MGST1) via the Keap1-Nrf2 pathway (92). This finding suggests that RSS orchestrate a broader antioxidant defense network beyond the canonical HO-1 axis. However, given that these observations were made in retinal pigment epithelial cells, further investigation is required to verify whether this specific mitochondrial protective mechanism is conserved in immune cells.

##### 3.1.2.4 Unresolved questions: pathway crosstalk and donor specificity

Critical gaps persist: How do NF-κB and Nrf2 pathways coordinate temporally, and what molecular features determine donor-specific cysteine targeting? Answering these through integrated temporal multi-omics and structure-based design will enable precision RSS therapeutics.

#### 3.1.3 Mitochondrial quality control and programmed cell death regulation

Beyond NF-κB and Nrf2 regulation, RSS coordinate macrophage functional remodeling through mitochondrial quality control, programmed cell death pathways, and mitogen-activated protein kinase (MAPK) signaling cascades. These mechanisms provide additional layers of regulation in sepsis-associated organ dysfunction, burn injuries, and metabolic liver diseases.

PINK1/Parkin-mediated mitophagy protects macrophage phenotypic homeostasis across diverse pathological contexts. In sepsis-associated cardiorenal syndrome type 5, exogenous NaHS and S-propargyl-cysteine (SPRC) activate this pathway to clear damaged mitochondria, restore membrane potential, suppress ROS generation, and promote RAW264.7 macrophage M1-to-M2 transition in cardiac/renal tissues (93). In acute arsenic exposure, endogenous PINK1/Parkin upregulation with elevated LC3-II/I ratio confers hepatoprotection; pharmacological PINK1 inhibition (cyclosporin A) exacerbates arsenic-induced hepatic macrophage inflammation (94). These findings establish mitophagy as a convergent mechanism linking exogenous H_2_S donors and endogenous stress responses to macrophage phenotypic homeostasis.

In rat burn wound models, NaHS modulates the expression of three major MAPK family members, including ERK, c-Jun N-terminal kinase (JNK) and p38, in basal skin macrophages at injury sites (95). Given that MAPK pathways govern macrophage inflammatory cytokine production, proliferation, and apoptosis decisions, this modulation pattern suggests RSS integrate pleiotropic immunoregulatory effects through fine-tuning MAPK pathway.

Indirect evidence for RSS involvement in metabolic liver disease emerges from studies on the immune checkpoint molecule Tim-3. In methionine-choline-deficient diet-induced non-alcoholic steatohepatitis (NASH), Tim-3 suppresses macrophage IL-1β and IL-18 secretion, thereby exerting hepatoprotection. Similarly, N-acetylcysteine (NAC), a cysteine derivative and ROS scavenger, ameliorates hepatic steatosis and inflammation (96). Although H_2_S levels were not directly measured in this study, NAC’s dual function as both a cysteine donor (H_2_S biosynthetic substrate) and ROS inhibitor implies potential crosstalk between sulfur metabolism and the ROS-inflammasome axis, warranting further investigation into RSS roles in metabolic liver pathology.

**Figure 3:**
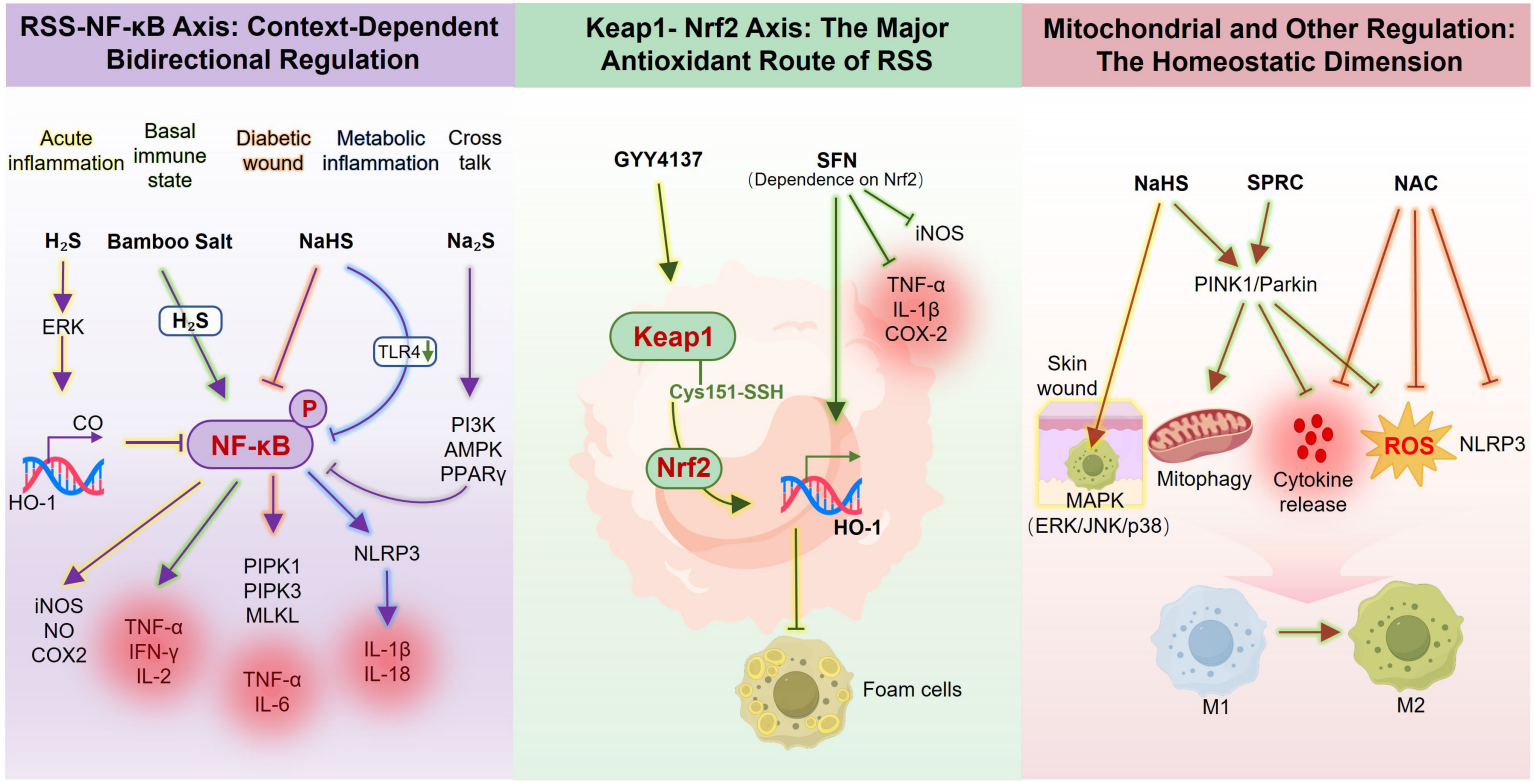
RSS modulate macrophage redox balance and inflammatory signaling: RSS, including H_2_S and its donors (NaHS, Na_2_S, GYY4137, SPRC), modulate macrophage activation through three interrelated axes. (Left) RSS–NF-κB axis: H_2_S suppresses NF-κB signaling via ERK–HO-1–CO pathway to reduce iNOS/NO production. In basal immune states, bamboo salt–derived H_2_S activates NF-κB to enhance TNF-α, IFN-γ, and IL-2 expression. In diabetic or metabolic inflammation, NaHS and Na_2_S inhibit NF-κB activation and NLRP3 inflammasome priming, and proinflammatory cytokine release through the TLR4 or PI3K/AMPK/PPARγ pathways. (Center) Keap1–Nrf2 axis: GYY4137 and sulforaphane (SFN) promote Keap1 persulfidation, thereby facilitating Nrf2 nuclear translocation and antioxidant gene expression through distinct mechanisms. (Right) Mitochondrial and redox regulation: NaHS and SPRC activate PINK1/Parkin-mediated mitophagy, and NAC indirectly scavenges ROS and inhibit cytokine release and NLRP3 activation. Collectively, these pathways restore redox homeostasis, promote M2 macrophage polarization, and facilitate inflammation resolution.

Collectively, RSS regulates macrophage function through mitochondrial quality control, MAPK signaling, and potential interactions with immune checkpoint-ROS pathways. These mechanisms complement NF-κB and Nrf2 (Sections 3.1.1-3.1.2), constituting a multilayered regulatory network (**Figure 3**). The therapeutic relevance of these mechanisms is further validated in disease models and innovative biomaterial systems, as examined in the following section.

#### 3.1.4 RSS-driven immunometabolic reprogramming: the glycolysis-mitochondria axis

In addition to maintaining mitochondrial integrity, RSS further regulate immune cell fate by controlling the metabolic switch between glycolysis and mitochondrial respiration.

H_2_S targets GAPDH, modifying it via persulfidation at the active site cysteine (Cys150). While initial studies reported that this modification significantly enhances GAPDH enzymatic activity due to the increased nucleophilicity of the persulfide group (97, 98), subsequent *in vitro* analyses utilizing polysulfide donors have suggested that persulfidation may instead inhibit catalytic function, possibly due to steric hindrance at the active site (99). This discrepancy suggests that the functional impact of GAPDH persulfidation may be context-dependent or sensitive to the specific sulfur donors employed.

A mechanistic breakthrough by Rahman *et al.* revealed that Mycobacterium tuberculosis infection triggers excessive host CSE expression, leading to supraphysiological H_2_S levels. Unlike its physiological role, this surfeit of H_2_S acts as a broad metabolic suppressor, inhibiting central carbon metabolism and mitochondrial respiration. The suppression of glycolytic flux is particularly detrimental, as it prevents the stabilization of Hypoxia-inducible factor 1-alpha (HIF-1α). As HIF-1α is required for IL-1β production and NADPH-dependent ROS generation, its destabilization leads to impaired bactericidal capacity (100). Consequently, CSE-deficient macrophages maintain robust glycolytic rates and HIF-1α levels, thereby exhibiting enhanced bacterial control. Our previous work showed that sulfonyl azide effectively scavenges H_2_S (101), providing a potential tool to modulate RSS levels. Thus, targeting RSS-producing enzymes or scavenging excess RSS to restore the glycolysis-HIF-1α axis may reverse metabolic immunosuppression.

#### 3.1.5 Disease model validation and therapeutic translation

The mechanistic insights from Sections 3.1.1-3.1.4 have been translated into therapeutic application across multiple metabolic disease models and innovative biomaterial platforms. Rather than reiterating individual studies, this section synthesizes disease-mechanism-intervention relationships and highlights emerging delivery technologies addressing the pharmacokinetic limitations of traditional H_2_S donors.

RSS-based interventions demonstrate therapeutic efficacy across diverse metabolic disease contexts (**Table 1**). In diabetic complications, endogenous H_2_S deficiency correlates with chronic NF-κB hyperactivation (wounds, osteoarthritis), impaired Nrf2 signaling (atherosclerosis), and defective mitophagy (nephropathy). Exogenous supplementation consistently restores macrophage homeostasis, thereby reducing tissue infiltration. In obesity-related inflammation, CSE deficiency in adipose tissue macrophages (ATMs) and perivascular adipose tissue (PVAT) drives systemic metabolic dysfunction, reversible by H_2_S application or CSE overexpression. Beyond metabolic disorders, targeting RSS-driven immunometabolism shows promise in infectious diseases.

**Table 1:** RSS-Based therapeutic interventions in the metabolic disease model.

Disease model	Defect	Intervention	Mechanism	Key outcomes	References
Diabetic wounds (*db/db* mice)	↓CSE/H_2_S, ↑NF-κB p65 phosphorylation	NaHS	NF-κB inhibition, ↓necroptosis	M1→M2 shift, ↑Wound closure, ↑Angiogenesis	(74, 87)
Diabetic osteoarthritis	↓H_2_S synthases, ↑M1 markers	GYY4137	↓HIF-1α, ↑HO-1	↓CD68^+^ infiltration, ↓Synovial inflammation	(102)
Diabetic atherosclerosis (STZ-LDLr^−^/^−^)	Oxidative stress	GYY4137	Keap1 Cys151 persulfidation→Nrf2 activation	↓Plaque formation, ↓ Foam cells, ↓ Superoxide anion	(89)
Obesity vasculopathy	↓vascular H_2_S (via PVAT iNOS)	GYY4137	PVAT M1 macrophages deplete vascular H_2_S	↑Endothelial dysfunction	(103)
Obesity metabolic inflammation	↓CSE in ATMs, ↑SOCE	NaHS or CSE overexpression	↓Ca^2+^ signaling, ↓NFAT/NF-κB	↓TNF-α/IL-6, improved insulin sensitivity	(85)
NASH (MCD diet)	Inflammatory moDC expansion	NaHS	Selective moDC inhibition	↓TNF-α^+^ CX3CR1^+^ moDCs, ↓Liver damage	(104)
Tuberculosis (Mtb infection)	↑Endogenous CSE/H_2_S, ↓Glycolysis, ↓HIF-1α	PAG (CSE inhibitor) H_2_S scavenges	Restoring glycolysis → HIF-1α stabilization → IL-1β/IL-12 production	↓Bacterial burden, ↑Host survival, ↑M1 immunometabolic function	(100, 101)

Traditional H_2_S donors (NaHS, Na_2_S) suffer from rapid release kinetics and poor spatiotemporal control. To overcome these pharmacological limitations, novel polysulfide delivery systems have emerged with superior efficacy. N-acetylcysteine polysulfide (NAC-S2), a cell-permeable sulfane sulfur donor distinct from traditional sulfide salts (105, 106), potently disrupts TLR4 signal and downstream cytokine storms in lethal endotoxin shock models, highlighting the pharmacodynamic advantages of polysulfide donors in managing systemic inflammation. Complementing these molecular advances, recent engineering innovations further address delivery constraints through three technological tiers: (1) Responsive nanocarriers: pH/ROS-triggered release systems, like iron sulfide nanocomposites GOx@FexSy/AZM for infected diabetic wounds (107) that match therapeutic action to microenvironmental cues; (2) Smart hydrogels: microenvironment-adaptive platforms, such as CoS-loaded silk fibroin hydrogels (108), GelMA-ODex@RRHD (109); (3) Multifunctional biomaterials: conductive hydrogels coupling H_2_S release with bioelectric signaling to synergistically enhance M2 polarization and tissue regeneration (e.g., PEDOT-alginate/gelatin systems) (110). These platforms demonstrate evolution from single-target intervention toward multidimensional, spatiotemporally precise regulation of macrophage function.

Despite promising preclinical efficacy, clinical translation faces critical challenges. First, long-term safety requires further characterization. Second, hormetic dose-response curves demand patient-stratified dosing based on endogenous RSS levels. Third, delivery routes (topical for wounds, inhalation for lung disease, or systemic) must align with disease pathology. Finally, non-invasive biomarkers for baseline RSS status and treatment monitoring remain undeveloped. Addressing these gaps will enable rational design of RSS-based precision therapeutics.

### 3.2 RSS regulation of neutrophil function and NETosis

Neutrophils, as core effector cells of innate immunity, play pivotal roles in host defense, inflammation regulation, and tissue repair. However, in metabolic diseases including diabetes, atherosclerosis, and hyperhomocysteinemia, neutrophils exhibit pathological hyperactivation characterized by excessive ROS generation, hyperactive chemotaxis, and aberrant neutrophil extracellular trap (NET) release. These dysregulated responses significantly exacerbate tissue damage and complications of metabolic diseases. H_2_S, as a representative RSS, demonstrates context-dependent bidirectional regulation of neutrophil function: suppressing over-activation under pathological conditions while preserving antimicrobial defense capacity. Understanding RSS-neutrophil interactions provides mechanistic rationale for developing RSS-based interventions in metabolic disease complications.

#### 3.2.1 Chemotaxis inhibition and inflammatory cytokine suppression

In type 1 diabetes mellitus (T1DM) rat models, serum IL-1β and IL-8 levels are significantly elevated, accompanied by upregulated neutrophil chemokine receptor CXCR2 expression, indicating diabetes-driven neutrophil activation. NaHS treatment reduces IL-1β and IL-8 expression and secretion while reversing CXCR2 overexpression, demonstrating that RSS inhibits neutrophil chemotactic responses (111). This receptor-level intervention represents a proximal control point for limiting neutrophil recruitment to inflamed tissues.

In LPS-induced acute lung injury (ALI) mouse models, pretreatment with the slow-release H_2_S donor GYY4137 significantly reduces neutrophil infiltration in lung tissue and inflammatory mediator accumulation in alveolar spaces, mechanistically linked to downregulated macrophage inflammatory protein-2 (MIP-2/CXCL2) and its receptor expression (112). *In vitro* experiments further confirmed that GYY4137 directly inhibits neutrophil transendothelial migration and MIP-2 release (112). Similarly, in bisphenol A (BPA)-induced lung injury, NaHS treatment significantly suppresses neutrophil infiltration into bronchoalveolar lavage fluid (BALF) (113), confirming the capacity to attenuate environmental toxicant-induced neutrophil accumulation. In granulocyte-macrophage colony-stimulating factor (GM-CSF)-induced differentiated HL-60 cells, NaHS significantly downregulates oncostatin M (OSM) expression by inhibiting the PI3K-Akt-NF-κB signaling pathway (114), further confirming RSS regulatory effects on neutrophil inflammatory cytokine profiles.

In mechanical ventilation-induced lung injury models, H_2_S demonstrates time-dependent protective effects with a relatively wide therapeutic window. Whether administered 1, 3, or 5 hours before ventilation (pretreatment) or at different time points after ventilation (post-treatment), H_2_S inhalation effectively reduces pulmonary edema, histological damage, neutrophil infiltration, and ROS generation (115). Mechanistically, H_2_S exerts pulmonary protection by synergistically inhibiting stress proteins, including heat shock protein 70 (HSP70), p38 MAPK phosphorylation, NADPH oxidase 2 (NOX2) expression, and ROS generation (116).

Clinical studies found that in asthma and chronic obstructive pulmonary disease (COPD) patients, sputum H_2_S levels significantly positively correlate with neutrophil proportions, with elevation consistent with neutrophil-dominated airway inflammatory responses (117, 118). This observation supports H_2_S as a potential biomarker for neutrophil-related airway inflammation, revealing its dual role: exerting anti-inflammatory protective effects in acute inflammation models while reflecting neutrophil-mediated inflammatory states in chronic airway diseases.

#### 3.2.2 Context-dependent modulation of neutrophil effector functions

RSS regulation of basic neutrophil effector functions exhibits marked complexity and donor specificity. In isolated human polymorphonuclear leukocytes (PMNLs), exogenous RSS donors show no significant effects on chemotaxis and phagocytosis but display donor-dependent differences in oxidative burst and apoptosis regulation. For oxidative burst, NaHS enhances phorbol myristate acetate (PMA)- or E. coli-induced responses, whereas diallyl trisulfide (DATS) and cysteine selectively inhibit E. coli’s burst without affecting PMA stimulation. Indeed, garlic constituents have been shown to potently attenuate free radical generation from neutrophils, likely by scavenging oxidizing agents (119). For apoptosis regulation, NaHS, diallyl disulfide (DADS), and cysteine reduce PMNL apoptosis, whereas GYY4137 induces apoptosis via mitochondrial intrinsic pathways (120).

These findings reveal that RSS regulation of neutrophil function exhibits significant donor specificity and context dependence. It primarily exerts anti-inflammatory effects under pathological conditions while sparing basal immune defense, underscoring the need for rational donor selection.

Notably, RSS demonstrates opposite effects in specific pathological contexts. In elastase-induced abdominal aortic aneurysm (AAA) models, sodium thiosulfate (STS, Na_2_S_2_O_3_), significantly enhances neutrophil infiltration in aneurysm walls, especially MMP9^+^ neutrophils, while also observing accumulation of c-KIT^+^ MPO^+^ pre-neutrophil clusters (121). Conversely, CSE-deficient mice (CSE^−^/^−^) exhibit reduced neutrophil infiltration and limited aneurysm expansion under identical AAA induction conditions (121), suggesting RSS may promote neutrophil chemotaxis or activation in this specific vascular pathology. This context-dependent duality underscores the importance of considering disease-specific microenvironments.

#### 3.2.3 Mechanistic basis of NETosis inhibition

NETosis is characterized by chromatin DNA release interwoven with granular proteins (MPO, NE, CitH_3_); it plays dual roles in immunity: pathogen capture versus tissue damage amplification (122, 123). In metabolic diseases, excessive ROS generation triggers pathological NETosis that becomes a key driver of complications. RSS inhibits NETosis through three convergent mechanistic axes that collectively suppress the ROS-MAPK-PAD4 signaling cascade underlying chromatin decondensation.

##### 3.2.3.1 Axis 1: direct ROS scavenging and MAPK pathway suppression

The core pathological mechanism linking metabolic disease to NETosis is ROS-driven PAD4 activation. In diabetic foot patients and LepR *db/db* mice, NETs levels correlate with delayed wound healing. Exogenous Na_2_S treatment reduces PAD4 and CitH_3_ expression while decreasing NETs component release (dsDNA, MPO, NE) to accelerate healing (4). Mechanistically, H_2_S suppresses chromatin citrullination through two parallel pathways: direct ROS scavenging, and blocking MAPK (ERK1/2 and p38) phosphorylation that drives PAD4 activity. This ROS-MAPK-PAD4 axis represents the convergent signaling cascade through which diverse pathological stimuli, including 2,4,6-trinitrobenzenesulfonic acid (TNBS) (124), phorbol 12-myristate 13-acetate (PMA), monosodium urate (MSU) crystals (125, 126), LPS (127), diabetes-associated hyperglycemia (128), and hypochlorous acid (HOCl) (129), ultimately trigger NETosis.

A critical mechanistic insight is that these stimuli share a common pathway: they enhance ROS production in neutrophils, which then activates MAPK signaling to upregulate PAD4. H_2_S intervention blocks this cascade at multiple nodes, explaining its broad protective efficacy across different pathological contexts. However, an unresolved question is whether H_2_S directly inhibits PAD4 enzymatic activity or solely acts upstream through ROS/MAPK suppression. Determining the precise molecular target would inform rational donor design for maximizing NETosis inhibition. Notably, beyond ROS-dependent pathways, the pH-sensitive H_2_S donor JK-1 inhibits aspirin-induced MPO activation and neutrophil infiltration in gastric mucosal injury models (130), confirming H_2_S’s broad tissue-protective effects by regulating MPO activity and NETs component release across multiple pathological contexts.

##### 3.2.3.2 Axis 2: platelet-neutrophil crosstalk disruption

In hyperhomocysteinemia, H_2_S exerts anti-NETosis effects through an unexpected mechanism: disrupting platelet-neutrophil interactions. Homocysteine-induced endothelial damage and ROS generation promote platelet P-selectin upregulation, which activates neutrophils to trigger NETs release. NaHS treatment alleviates endothelial injury (decreased Bax/Bcl-2 ratio) and inhibits platelet p38 MAPK phosphorylation and P-selectin expression, reducing serum dsDNA concentration and NETs formation (131).

Further mechanistic dissection reveals that H_2_S blocks the platelet-derived danger signal HMGB1. By inhibiting platelet HMGB1 release, H_2_S prevents neutrophil TLR4/p38 MAPK axis activation and reduces ROS levels by upregulating SOD activity, thereby suppressing PAD4 expression (132). This platelet-targeting mechanism provides a distinct therapeutic angle beyond direct neutrophil effects, particularly relevant for thrombotic complications where platelet-NETs interactions drive pathology.

##### 3.2.3.3 Axis 3: epigenetic regulation via miRNA-mediated pathway

Beyond acute signaling inhibition, H_2_S exerts sustained anti-NETosis effects through epigenetic mechanisms. In neutrophils, H_2_S exposure upregulates miR-16-5p, which subsequently suppresses its downstream targets PIK3R1 and RAF1. Through this miRNA-mediated regulatory network, H_2_S multilevel blocks NETosis by: inhibiting PI3K/Akt and ERK signaling pathways, reducing respiratory burst levels, and attenuating IP3R-mediated ER calcium efflux and autophagy processes (133). This epigenetic layer provides a mechanistic explanation for sustained H_2_S effects that persist beyond immediate donor exposure.

**Figure 4:**
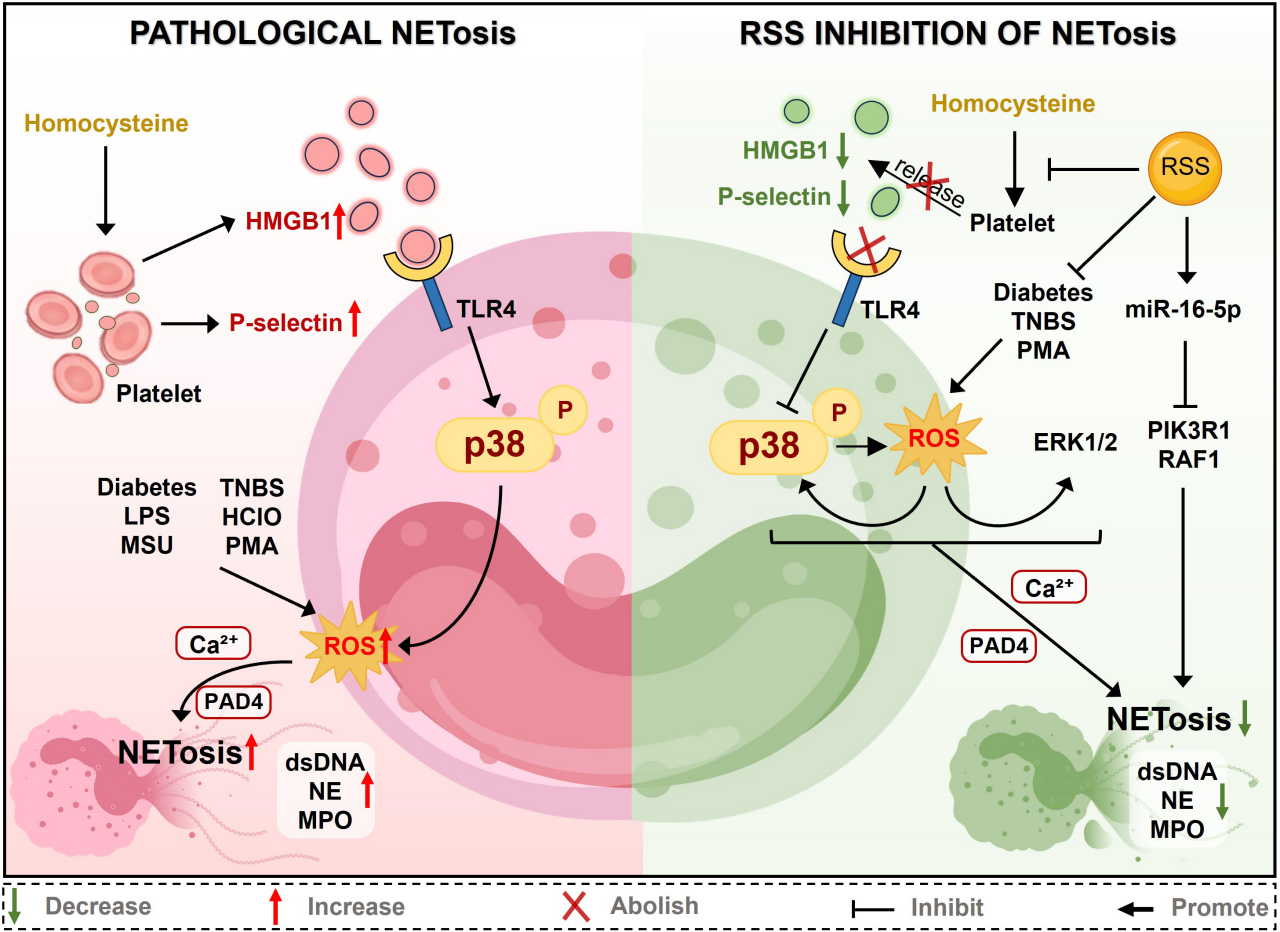
Schematic mechanism of pathological NETosis and its inhibition by RSS: (Left) Pathological NETosis is triggered by factors such as homocysteine, which activates platelets to release HMGB1 and P-selectin, subsequently activating the TLR4-p38 pathway. Pathological stimuli (e.g., Diabetes, LPS) and p38 signaling synergistically promote ROS generation. Activation of the p38 and ROS pathways converges on the Ca²^+^/PAD4 axis, ultimately driving NETosis and the release of dsDNA, NE, and MPO. (Right) RSS block this cascade by inhibiting platelet activation, reducing HMGB1/P-selectin, and suppressing ROS production. Furthermore, RSS inhibits the ERK1/2 pathway by promoting miR-16-5p. Through comprehensive suppression of the p38, ROS, and ERK1/2 signaling pathways, RSS effectively prevents Ca^2+^/PAD4 activation, thereby inhibiting NETosis.

Collectively, these three axes converge on a unified therapeutic model (**Figure 4**): H_2_S suppresses pathological NETosis through immediate ROS-MAPK inhibition (Axis 1), intercellular danger signal disruption via platelet-neutrophil crosstalk blockade (Axis 2) and sustained epigenetic reprogramming through miR-16-5p upregulation (Axis 3). This mechanistic stratification reveals context-dependent therapeutic opportunities: diabetic wounds primarily involve Axis 1 ROS dysregulation; thrombotic disorders engage Axis 2 platelet interactions; and drug-induced gastrointestinal injury responds to pH-sensitive donors. However, critical translational gaps persist: Does H_2_S directly persulfidate PAD4’s catalytic cysteines? What temporal kinetics govern miR-16-5p epigenetic memory? Can donor pharmacokinetics be matched to axis-specific requirements, such as fast-release donors (NaHS) for acute ROS scavenging versus slow-release donors (GYY4137) for sustained epigenetic effects? Addressing these through mechanistic target validation, temporal profiling, and systematic donor-mechanism matching studies will enable rational clinical translation from this multilevel regulatory framework.

#### 3.2.4 Disease model validation and translational prospects

In diabetic wound healing models, CSE expression downregulation and decreased H_2_S generation are closely associated with abnormally elevated NETs levels. Both serum from diabetic foot patients and wound tissues from *db/db* mice show significantly elevated NETs markers, accompanied by delayed wound healing. Exogenous Na_2_S or NaHS downregulates PAD4, CitH_3_, and NETs component (dsDNA, MPO, NE) release, reduces neutrophil infiltration, and promotes wound healing (4, 87). Proteomic analysis of human surgical wound exudates confirms enrichment of neutrophils and oxidative products (including MPO) in the wound microenvironment (134), providing clinical evidence supporting RSS intervention.

Nutritional intervention studies demonstrate that combined supplementation with vitamin D and L-cysteine (H_2_S precursor) reduces neutrophil/lymphocyte ratio and inflammatory marker levels (135). In diabetic peripheral neuropathy models, vitamin D exerts neuroprotective effects by restoring the CBS/H_2_S system (136). These findings suggest that enhancing endogenous H_2_S generation through nutritional intervention may improve inflammatory status in metabolic disease patients, providing non-pharmacological strategies for clinical application.

Novel H_2_S delivery systems provide technical foundations for clinical translation. pH-sensitive donors (JK-1), slow-release donors (GYY4137), and mitochondria-targeted donors (AP39) (112, 130, 137) have demonstrated the capacity to inhibit neutrophil over-activation or/and NETosis in multiple disease models, including diabetic wounds, acute lung injury, and gastric mucosal injury. These controlled-release platforms address the limitations of traditional fast-release donors by achieving spatiotemporally precise H_2_S delivery matched to pathological microenvironments, laying groundwork for clinical applications in metabolic disease complications.

Clinical translation requires addressing several key challenges. Donor specificity: the differential effects of NaHS, DATS, and GYY4137 on oxidative burst and apoptosis necessitate rational donor selection based on therapeutic goals. Context dependency: pro-infiltration effects observed in AAA models contrast with anti-inflammatory effects in lung injury, demanding disease-specific risk-benefit assessments. Biomarker validation: while sputum H_2_S correlates with neutrophil proportions in COPD patients, this relationship requires prospective validation as a therapeutic monitoring tool. Finally, combination strategies should be explored to integrate H_2_S manipulation (using both donors and scavengers) with existing anti-inflammatory therapies such as corticosteroids, potentially achieving synergistic neutrophil regulation.

### 3.3 RSS metabolic network regulation of dendritic cell function

Dendritic cells (DCs), serving as critical bridges connecting innate and adaptive immunity, exhibit functional plasticity regulated not only by classical cytokines but also by the cellular redox microenvironment. The RSS metabolic network, including H_2_S biosynthetic enzymes (CBS, CSE, 3-MST), the cysteine-GSH metabolism, and protein persulfidation, collectively modulates DC differentiation, maturation, and antigen presentation. Dysregulation of this network is associated with DC functional disorders in autoimmune diseases, metabolic inflammation, and tumor immune evasion.

#### 3.3.1 The RSS-cysteine-GSH metabolic axis as a regulatory triad

RSS regulation of DC function operates primarily through control of cysteine availability and GSH homeostasis, rather than solely through H_2_S signaling. This mechanistic insight emerges from studies showing that the cystine/glutamate antiporter (system xc^−^, xCT) serves as the gatekeeper of cellular sulfur metabolism (77, 138). System xc^−^ controls cystine uptake and its subsequent reduction to cysteine, the shared substrate for both GSH synthesis and H_2_S biosynthesis, creating a metabolic branch point where RSS production and antioxidant defense compete for limited cysteine pools. Blocking system xc^−^-mediated cystine transport creates a dual metabolic crisis: depleted GSH compromises antioxidant defense, while reduced cysteine pools limit endogenous H_2_S generation and protein persulfidation. The downstream consequences reveal this axis as a critical redox checkpoint: significantly reduced intracellular GSH levels severely disrupt monocyte-to-DC differentiation without affecting LPS-induced phenotypic maturation, significantly impair exogenous antigen presentation to MHC class II-restricted T cells, and completely block MHC class I cross-presentation (139). These findings establish that GSH homeostasis regulates stage-specific DC differentiation, with GSH and RSS biosynthesis sharing the same cysteine pool.

Beyond basal redox balance, the GSH-RSS axis integrates environmental danger signals through the Keap1/Nrf2 pathway. In human CD34-derived DCs and THP-1 cells, contact sensitizers (nickel, 1-chloro-2,4-dinitrobenzene, cinnamaldehyde) specifically induce Nrf2 target genes (*HMOX1, NQO1*) at early time points. Pretreatment with NAC significantly inhibits sensitizer-induced NQO1 expression and CD86 upregulation (140), indicating that GSH or RSS levels affect Keap1/Nrf2 activation and directly correlates with DC maturation status. This reveals the cysteine-GSH-RSS metabolic network as an adaptive defense system sensing environmental danger signals.

Critically, RSS-producing enzymes exert non-H_2_S-dependent DC regulation via direct cysteine pool control. In rat cardiac allograft models, the CSE inhibitor propargylglycine (PPG) delays rejection and selectively inhibits Th1 factors (T-bet, IL-12, IFN-γ) without affecting antibodies. Mechanistically, CSE inhibition acts directly on monocytes and DCs to reduce IL-12 production capacity through intracellular cysteine pool regulation, independent of NF-κB signaling or H_2_S (141). This indicates CSE’s non-canonical function: directly regulating DC immune programming via cysteine availability rather than H_2_S signaling. CSE and system xc^−^ constitute a dual-valve system governing intracellular cysteine/GSH homeostasis during DC differentiation. However, the molecular mechanisms linking cysteine availability to IL-12 transcriptional control remain undefined.

#### 3.3.2 H_2_S-mediated direct regulation in pathological contexts

Beyond cysteine pool control, H_2_S donors selectively inhibit pathological DC subsets in disease-specific contexts. In methionine/choline-deficient diet-induced metabolic dysfunction-associated steatohepatitis (MASH), inflammatory monocyte-derived DCs (moDCs) characterized by dual Ly6C^+^CD11b^+^CD11c^+^MHC-II^+^ expression and continuous TNF-α production undergo 4-fold expansion during late disease. NaHS specifically prevents TNF-α-producing CX3CR1^+^ moDC accumulation, reducing hepatic TNF-α levels and improving transaminase release without affecting normal liver macrophages (104). This selective targeting of pathological subsets reveals the precision immunoregulatory potential of RSS, yet the molecular determinants of this selectivity remain unidentified, including specific metabolic vulnerabilities or surface receptor profiles.

In CNS autoimmune diseases, GYY4137 reshapes DC cytokine balance toward immune tolerance. In CNS antigen-immunized mice, GYY4137 enhances TGF-β expression/secretion in DCs while reducing T cell-derived IFN-γ and IL-17 in lymph nodes and spinal cords. Critically, PBMC from multiple sclerosis patients exhibit significantly lower 3-MST expression compared to healthy controls, with 3-MST levels negatively correlating with pro-inflammatory factors (142), suggesting endogenous H_2_S deficiency promotes autoimmune pathology by weakening DC tolerogenic programs. GYY4137’s slow-release kinetics enable sustained low-concentration exposure, potentially more suitable for inducing DC reprogramming compared to fast-release donors.

An unresolved paradox is that H_2_S biological effects depend highly on source, chemical form, and concentration. Intestinal microbial protein fermentation-produced H_2_S exhibits cytotoxicity to bone marrow-derived DCs at high concentrations *in vitro*, with unclear effects on cytokine secretion at physiological ranges (143). This contrasts sharply with exogenous donor anti-inflammatory effects, suggesting rapid free H_2_S fluctuations produce non-specific toxicity whereas controllable release achieves targeted regulation.

#### 3.3.3 Dual roles of RSS in tumor immunity: therapeutic and pathological effects

In tumor microenvironments, RSS exhibit profound concentration- and source-dependent duality: exogenous RSS enhance anti-tumor immunity, whereas endogenous CBS/CSE overexpression in tumor cells creates immunosuppression. Understanding this paradox is critical for RSS-based cancer immunotherapy.

**Figure 5:**
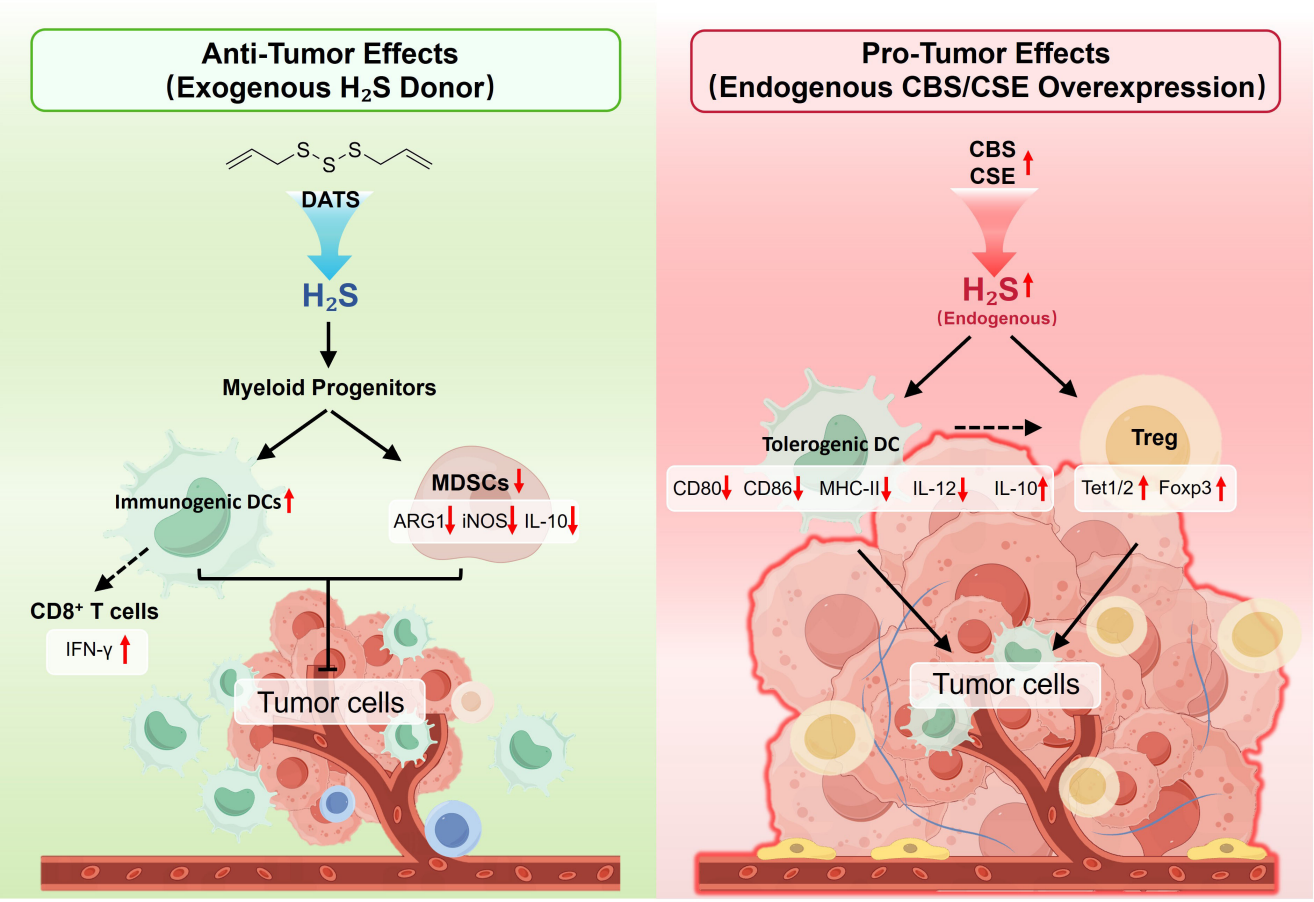
RSS-mediated bidirectional regulation of dendritic cell function in the tumor microenvironment. (Left) Anti-tumor effects: Exogenous DATS modulates myeloid progenitor differentiation, promoting immunogenic DCs while suppressing myeloid-derived suppressor cells (MDSCs). Immunogenic DCs indirectly activate CD8^+^ T cells with enhanced IFN-γ production, leading to effective tumor elimination. (Right) Pro-tumor effects: Endogenous CBS/CSE overexpression increases H_2_S production, generates tolerant DCs and promotes regulatory T cells, with decreased co-stimulatory molecules and altered cytokine profile. The formation of immunosuppressive tumor microenvironment promotes tumor progression.

Exogenous donors remodel tumor immunosuppression by targeting myeloid-derived suppressor cells (MDSCs). In B16F10 melanoma models, DATS treatment significantly inhibits tumor growth through selective MDSC frequency reduction in spleen, peripheral blood, and tumor tissues, accompanied by increased tumor-infiltrating CD8^+^ T cells and DCs with restored T cell proliferation. Mechanistically, DATS upregulates the expression of antioxidant genes (*GCLC, GCLM, HMOX*), suggesting that local antioxidant system modulation indirectly promote DC maturation by relieving MDSC-mediated suppression (144) (**Figure 5A**).

Conversely, elevated CBS/CSE expression in tumor cells correlates with poor prognosis and immune evasion. In ovarian cancer tissues, both CBS and CSE are significantly upregulated, with high CBS expression correlating with shorter progression-free survival (PFS, *P* = 0.02) and overall survival (OS, *P* = 0.03). Immune infiltration analysis reveals that CBS/CSE expression significantly correlates with CD4^+^ T cell, neutrophil, macrophage, and DC infiltration scores, suggesting tumor-derived sulfur fosters immunosuppressive milieus (145). In breast cancer, CBS/CSE/3-MST overexpression associates with immunosuppressive phenotypes; inhibiting these enzymes suppresses tumor viability/migration while modulating immunoregulatory molecule expression (GAL3/9, MICA/B, CD155) (146). In tumor microenvironments, tolerogenic DCs are characterized by low CD80/CD86/MHC-II expression, reduced IL-12, and increased IL-10 production, favoring Treg expansion over effector T cell activation (147–149) (**Figure 5B**).

Mechanistically, endogenous RSS remodels immunosuppression through multiple pathways. They promote Tet1/Tet2 expression by sulfhydrating transcription factor NFYB, catalyzing 5mC to 5hmC conversion at the Foxp3 locus to stabilize Treg differentiation (150). CBS/CSE overexpression in cisplatin-resistant OVCAR8 cells (145) suggests dysregulated RSS metabolism promotes tumor progression through enhanced Treg differentiation, impaired DC maturation, and immunosuppressive microenvironment maintenance.

Comparative analysis reveals this duality arises from four mechanistic distinctions (**Figure 5**): (1) Temporal kinetics—exogenous donors produce high peak concentrations with short durations possibly preferentially targeting MDSC redox sensitivity, whereas endogenous enzymes continuously produce H_2_S enabling chronic immune reprogramming. (2) Spatial distribution—exogenous RSS distributes uniformly throughout tumor microenvironments, whereas endogenous sulfur enriches around tumor cells, forming local immunosuppressive gradients. (3) Target cell selectivity—DATS’s selective MDSC inhibition may stem from MDSC metabolic vulnerabilities, while tumor cell-released H_2_S affects multiple immune cells via paracrine mechanisms. (4) Dose-response relationships—RSS effects on immune cells may follow hormetic curves, with low-to-moderate concentrations promoting immunosuppression while high-concentration pulsatile exposure produces cytotoxicity or reprogramming effects.

#### 3.3.4 Translational implications and mechanistic uncertainties

RSS regulation of DC function reveals a multi-tiered control system: the cysteine-GSH-RSS metabolic axis serves as a foundational redox checkpoint governing DC differentiation (Section: 3.3.1), H_2_S signaling provides context-dependent modulation of pathological DC subsets and tolerogenic programming (Section: 3.3.2), and tumor contexts reveal source- and concentration-dependent dual RSS roles (Section: 3.3.3).

However, critical mechanistic gaps constrain clinical translation: First, what mechanisms link cysteine availability to DC transcriptional programs? CSE inhibition reduces DC IL-12 production independent of H_2_S (141), yet the cysteine-responsive transcription factors or epigenetic modifiers remain unidentified. Metabolomic profiling of cysteine-depleted DCs coupled with transcriptomic analysis could reveal these regulatory nodes.

Second, what determines donor selectivity for pathological versus physiological DC subsets? H_2_S selectively targets CX3CR1^+^ moDCs in NASH without affecting normal macrophages (104), but the molecular basis is undefined, including metabolic signatures, surface receptors, or redox states. Single-cell multi-omics comparing donor-responsive versus resistant DC populations could identify selectivity determinants.

Third, does the RSS-GSH axis apply universally across DC subsets? Different DC populations (cDC1, cDC2, pDCs, moDCs) exhibit distinct metabolic programs, yet subset-specific RSS profiling is lacking. Such data would enable precision targeting of pathogenic DCs in autoimmune and tumor contexts.

## 4 RSS and adaptive immunity

Beyond innate immunity, RSS metabolic networks also profoundly shape adaptive immune responses. DCs provide the critical link: DC-derived signals directly program T and B cell differentiation and function. RSS regulation extends beyond innate immunity to control T cell responses, Treg stability, CD8^+^ T cell metabolism, and antibody function. This establishes RSS as key coordinators linking metabolism to adaptive immunity.

### 4.1 RSS regulation of T cell activation and functional homeostasis

#### 4.1.1 Autocrine H_2_S enhancement of T cell activation

**Figure 6:**
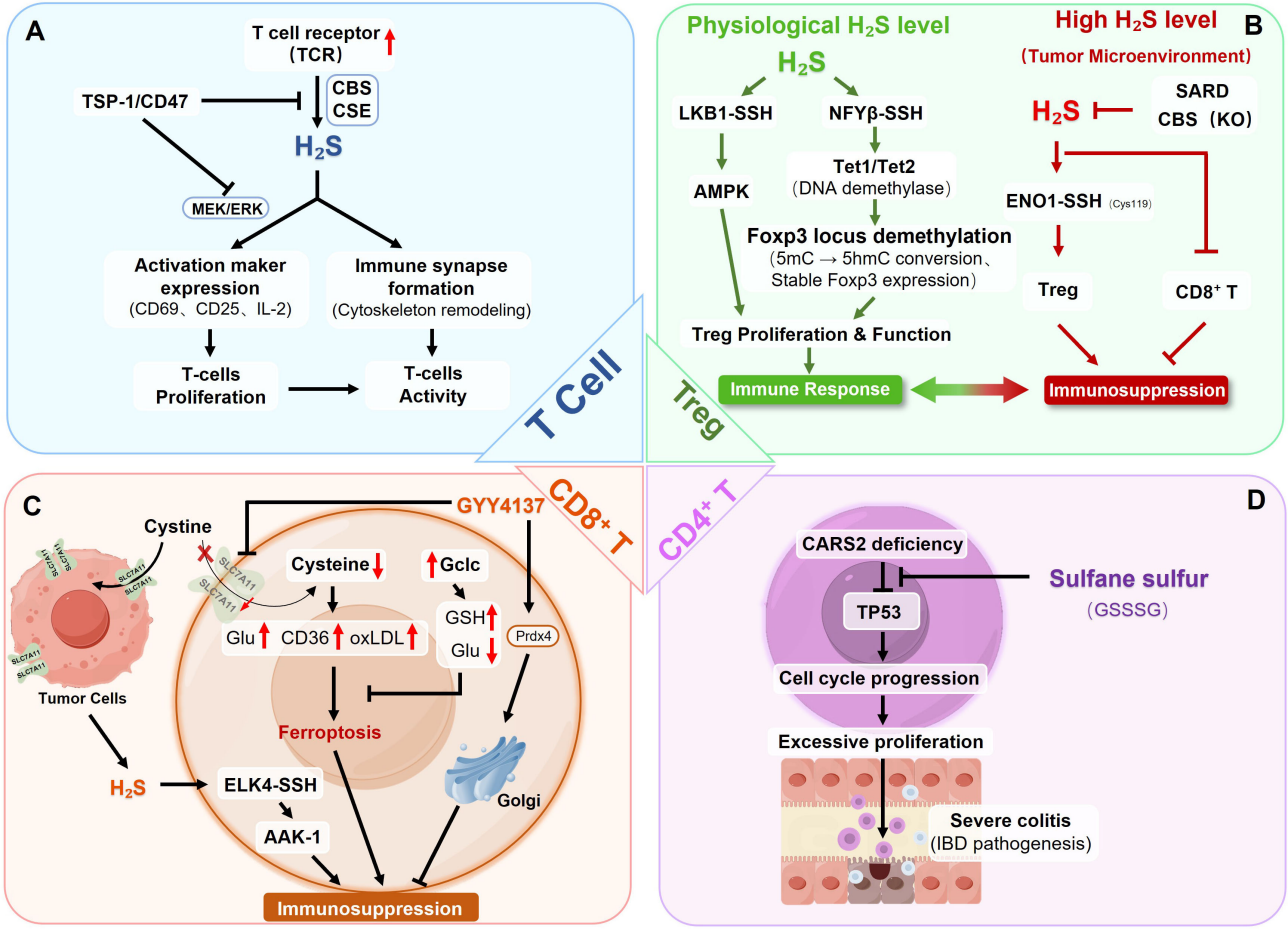
Multifaceted roles of RSS in adaptive immune regulation: **(A)** H_2_S acts as a positive regulator in T-cell activation. TCR stimulation upregulates CBS and CSE. The produced H_2_S, through the MEK/ERK pathway, promotes immune synapse formation, leading to increased activation marker expression (CD69, CD25, IL-2), T-cell proliferation, and overall T-cell activity. **(B)** H_2_S exerts a dual, concentration-dependent role in Treg cell function. At physiological levels, H_2_S maintains Treg proliferation and function, ensuring a balanced immune response. This is mediated by persulfidation of LKB1 (activating AMPK) and NFYB (activating Tet1/Tet2), to promote Foxp3 locus demethylation. Conversely, high H_2_S levels, characteristic of the tumor microenvironment, drive immunosuppression by enhancing Treg function (via ENO1-SSH) while directly inhibiting CD8^+^ T-cell activity. **(C)** Tumor-derived H_2_S suppresses CD8^+^ T cell migration and anti-tumor immunity via the ELK4-SSH/AAK-1 pathway. Conversely, exogenous H_2_S donors protect CD8^+^ T cells by preserving Golgi integrity through Prdx4 shuttling. Additionally, cystine availability governs CD8^+^ T cell ferroptosis resistance: cystine deprivation triggers the glutamate-CD36-lipid peroxidation cascade, while Gclc overexpression enhances GSH synthesis and consumes excess glutamate to suppress CD36-mediated ferroptosis, thereby maintaining CD8^+^ T cell effector function. **(D)** CARS2 deficiency leads to TP53 downregulation, which promotes cell cycle progression and enhances CD4^+^ T-cell excessive proliferation, contributing to the pathogenesis of severe colitis. GSSSG inhibits this pathological cascade, possibly by suppressing the excessive proliferation, though the precise mechanism remains to be determined.

H_2_S plays an important autocrine regulatory role in T cell activation. Upon T cell receptor (TCR) stimulation, T cells rapidly upregulate expression of endogenous H_2_S synthases (CBS and CSE), thereby enhancing endogenous H_2_S generation (151). Within physiologically relevant nanomolar concentration ranges, H_2_S significantly enhances TCR-mediated T cell activation, manifested by upregulation of early activation markers CD69 and CD25, increased IL-2 expression, and enhanced proliferative capacity. Silencing CBS and CSE expression impairs T cell activation and proliferation, while exogenous Na_2_S supplementation completely rescues this defect, confirming the necessity of endogenous H_2_S in T cell activation (6) (**Figure 6A**).

At the molecular level, H_2_S optimizes immunological synapse formation efficiency by promoting actin cytoskeleton dynamic rearrangement and microtubule-organizing center (MTOC) reorientation, thereby enhancing TCR signal transduction (152). Additionally, H_2_S promotes downstream signaling cascades by enhancing MEK-dependent ERK phosphorylation (153). Notably, due to limited mitochondrial oxidative metabolism of H_2_S under hypoxic conditions, its stimulatory activity on T cells is further enhanced in low oxygen environments (152), suggesting potential relevance in hypoxic tumor microenvironments or inflamed tissues.

H_2_S’s immunostimulatory effects are strictly constrained by endogenous inhibitory mechanisms. Thrombospondin-1 (TSP-1) functions as the first discovered endogenous H_2_S signal inhibitor by binding its receptor CD47 (153). The TSP-1/CD47 signaling axis limits H_2_S’s pro-activation effects through two key mechanisms: inhibiting TCR-induced upregulation of CBS and CSE; blocking H_2_S-enhanced ERK phosphorylation (153). T cells from TSP-1 knockout mice exhibit heightened sensitivity to H_2_S-dependent activation, while exogenous TSP-1 inhibits H_2_S responses in wild-type but enhances responses in CD47 knockout T cells, demonstrating CD47’s necessity and sufficiency for TSP-1-mediated H_2_S inhibition (152, 153). This precise regulatory balance ensures appropriate T cell activation, preventing excessive immune activation.

RSS-induced immunoregulatory effects exhibit bidirectionality and context dependence. In asthma mouse models, CSE expression levels in lung tissue are closely associated with airway inflammation and hyperresponsiveness. In ovalbumin (OVA)-induced asthma models, airway inflammation accompanies downregulated endogenous H_2_S generation, while exogenous H_2_S donor supplementation significantly inhibits airway hyperresponsiveness, reduces inflammatory cell infiltration, decreases Th2-type cytokine (IL-4, IL-5, IL-13) expression, and ameliorates airway remodeling (154). This anti-inflammatory effect relates to inhibition of NF-κB-mediated inflammatory and oxidative responses. Furthermore, H_2_S alleviates allergic airway hyperresponsiveness by regulating mast cell activation (155). These findings reveal RSS complex regulatory network in different immune contexts: promoting T cell activation in anti-infection immunity while exerting inflammation-suppressing and tissue-protecting effects in excessive immune states.

#### 4.1.2 RSS-mediated regulation of Treg differentiation and functional stability

H_2_S has been shown to play a crucial role in maintaining Treg-mediated immune homeostasis through epigenetic regulation of the Foxp3 locus. Stable differentiation of Foxp3^+^ Treg cells relies on specific hypomethylation patterns at the Foxp3 locus, which are established in part through H_2_S-mediated regulation of the DNA demethylases Ten-eleven translocation (Tet) 1 and Tet2. At the molecular level, H_2_S promotes persulfidation of the nuclear transcription factor NFYβ, enhancing its binding to the promoters of Tet1 and Tet2 and thereby sustaining the expression of these demethylases. Under synergistic TGF-β and IL-2 signaling, activated Smad3 and Stat5 recruit Tet1 and Tet2, respectively, to the Foxp3 locus. These enzymes catalyze the conversion of 5-methylcytosine (5mC) to 5-hydroxymethylcytosine (5hmC), thereby establishing Treg-specific hypomethylation patterns that stabilize Foxp3 expression. Consistently, dual deletion of Tet1 and Tet2 results in Foxp3 hypermethylation, impaired Treg differentiation and function, and ultimately the development of systemic autoimmune disease (150).

The immunoprotection of CSE/H_2_S in CD4^+^ T cells are confirmed in hypertension models. In peripheral blood lymphocytes from hypertensive patients and spontaneously hypertensive rats, CSE-induced H_2_S generation is significantly reduced, negatively correlating with blood pressure but positively correlating with serum IL-10 levels. T cell-specific CSE knockout mice exhibit 5–8 mmHg baseline blood pressure elevation and exacerbated angiotensin II-induced hypertension, accompanied by impaired mesenteric artery dilation and arterial inflammation attributed to reduced Treg numbers in blood and kidneys, leading to excessive CD4^+^/CD8^+^ T cell infiltration into perivascular adipose tissue and kidneys (156). Mechanistically, H_2_S produced by CSE activates downstream AMPK signaling by inducing persulfidation of liver kinase B1 (LKB1), which enhances its substrate-binding capacity and promotes Treg differentiation and proliferation. Adoptive Treg transfer to CSE knockout mice reverses hypertension, vascular dysfunction, and immune infiltration, demonstrating that CSE deficiency-induced hypertension is partly mediated by reduced Treg numbers and subsequent vascular/renal immune inflammation (156).

Notably, the engagement of the AMPK node by RSS exhibits profound context-dependency. In contrast to this LKB1-mediated homeostatic support in Tregs, our recent work in hepatocellular carcinoma reveals that the exogenous polysulfide donor PSCP activates AMPK via a distinct metabolic route: it competitively binds to the mitochondrial Complex I subunit Ndus3, triggering an ATP crisis that subsequently activates the AMPK-p53 axis to induce tumor cell apoptosis (39). This demonstrates that RSS can dictate opposing cell fates—survival versus death—by engaging the same kinase (AMPK) through divergent molecular mechanisms (direct enzymatic persulfidation versus indirect metabolic stress).

Conversely, endogenous RSS accumulation within the tumor microenvironment can hijack metabolic machinery to drive immunosuppression. In colorectal cancer, elevated H_2_S levels promote differentiated CD4^+^CD25^+^Foxp3^+^ Treg activation by sulfhydrating enolase 1 (ENO1 Cys119), constructing immunosuppressive microenvironments. CBS heterozygous knockout (CBS^+^/^−^) or sulfur-amino acid restricted diet (SARD) reduces tumor Treg numbers, increases CD8^+^ T cell/Treg ratios, and enhances anti-PD-L1/anti-CTLA-4 immune checkpoint blockade efficacy (157). This finding supports engineered microbial-nanoparticle hybrid (E.coli@Cu_2_O) therapeutic strategies. This system consumes endogenous H_2_S at tumor sites, converting Cu_2_O to Cu_x_S, thereby weakening the tumor’s immunosuppressive shield while enhancing ferroptosis and cuproptosis, which significantly promotes DC maturation and CD8^+^ T cell activation (158).

In summary, this dual role highlights that physiological H_2_S promotes Treg differentiation via epigenetic (Tet1/Tet2) and metabolic (LKB1-AMPK) pathways to maintain immune homeostasis, whereas excessive tumor-derived H_2_S over-activates Tregs through ENO1 persulfidation, driving pathological immunosuppression (**Figure 6B**).

#### 4.1.3 Metabolic and redox regulation of CD8^+^ T cell survival and function

RSS exerts multilevel regulatory effects in CD8^+^ T cell-mediated anti-tumor immunity. In sickle cell disease (SCD), a genetic blood disorder, the CD8^+^ T cell three-dimensional genomic structure undergoes rearrangement, causing impaired chromosomal interactions between the anti-ferroptosis gene SLC7A11 and H_2_S biosynthesis-related genes, with downregulated expression making CD8^+^ T cells more susceptible to ferroptosis, weakening anti-tumor immune function, and promoting tumor growth (14). Studies show that in mouse and humanized SCD models, restoring H_2_S levels rescues SLC7A11 expression, reduces ferroptosis, and enhances anti-tumor immune responses.

In the tumor microenvironment (TME), preferential cystine uptake by tumor cells due to metabolic reprogramming to highly express cystine/glutamate transporter (SLC7A11) has been reported (159). Cystine deprivation causes metabolic stress on tumor-infiltrating CD8^+^ T cells, activating the glutamate-CD36-lipid peroxidation cascade that drives ferroptosis (160). Cystine deficiency disrupts GSH synthesis while interfering with cystine/glutamate exchange, leading to intracellular glutamate accumulation that upregulates scavenger receptor CD36, promoting excessive oxidized lipid uptake and lipid peroxidation. This ferroptotic program manifests as impaired memory formation, loss of stemness, exhaustion marker (PD-1, TIM-3) upregulation, and reduced effector cytokine (IFN-γ, TNF-α, IL-2) secretion, severely weakening anti-tumor immunity (161). In rectal cancer tissues, H_2_S level is significantly elevated. It increases the expression of AAK-1 through the persulfidation of cysteine residues in ETS domain-containing protein Elk-4 (ELK4), thereby inhibiting CD8^+^ T cell migration and immunity (157).

Glutamate-cysteine ligase catalytic subunit (Gclc) overexpression confers protection through dual mechanisms: enhancing glutathione synthesis (combating oxidative stress) and consuming excess glutamate (blocking CD36 upregulation), comprehensively resisting cystine deprivation-induced ferroptosis and exhaustion. In adoptive cell therapy, engineered Gclc-expressing CD8^+^ T cells demonstrate superior tumor control, reduced ferroptosis, and enhanced memory phenotype in melanoma models (162). This reveals how cysteine availability indirectly regulates CD8^+^ T cell ferroptosis sensitivity and anti-tumor function through glutathione-mediated redox balance, as cysteine serves as both a precursor for H_2_S synthesis and a rate-limiting substrate for glutathione production.

Beyond ferroptosis resistance, RSS signaling protects CD8^+^ T cells by maintaining organelle integrity. Metabolic disorders and oxidative stress in the tumor microenvironment impair Golgi apparatus function, causing Golgi stress and protein modification/transport disorders that inhibit T cell function. Studies show that under TME stress, Golgi structure (e.g., GM130 expression) declines, and this structural disruption is reversed by GYY4137 or CBS overexpression (163). Such interventions enhance T cell stemness, antioxidant capacity, and protein translation, partly through Prdx4 shuttling between ER and Golgi. In melanoma and lymphoma models, CD8^+^ T cells with such interventions exhibit superior tumor control after adoptive transfer (**Figure 6C**).

#### 4.1.4 Emerging reactive persulfide and polysulfide pathways in CD4^+^ T cell regulation

Beyond H_2_S, other RSS forms, including cysteine persulfide (CysSSH) and glutathione trisulfide (GSSSG), exert regulatory effects on T cell immunity (164, 165). These RSS are generated by cysteinyl-tRNA synthetases (CARS) via their cysteine persulfide synthase (CPERS) activity. Both cytosolic CARS1 and mitochondrial CARS2 contribute to cellular reactive persulfide and polysulfide pools, though with distinct subcellular localization and functional implications (166). CARS1-derived cytosolic RSS may preferentially modulate cytoplasmic signaling pathways and translation-related processes, while CARS2-generated mitochondrial RSS likely influence mitochondrial metabolism and bioenergetics. Collectively, these enzymes maintain cellular reactive persulfide and polysulfide homeostasis, which exerts endogenous proliferation-inhibiting effects in CD4^+^ T cells (5), representing a distinct regulatory mechanism from H_2_S’s pro-activation effects (Section 4.1.1) and highlighting RSS family functional diversity.

CARS2/CPERS-dependent RSS metabolism critically limits CD4^+^ T cell proliferation in a cell-intrinsic manner in inflammatory bowel disease (IBD). Under homeostatic conditions, Cars2^+^/^−^ mice exhibit spontaneous effector/memory CD4^+^ T cell accumulation in the colon with aging; under lymphopenic conditions, Cars2^+^/^−^ CD4^+^ T cells show enhanced cell cycle entry with decreased Trp53 expression, triggering severe colitis that is rescued by GSSSG supplementation. Reanalysis of human colon CD4^+^ T lymphocyte datasets confirms that CARS2 downregulation associates with IBD pathogenesis, with GSSSG inhibiting human CD4^+^ T cell proliferation *in vitro* (5). Together, these findings demonstrate that CARS2/CPERS-dependent RSS metabolism is essential for homeostasis of intestinal effector/memory CD4^+^ T cells, and that dysregulation of this metabolic pathway can lead to development of gut inflammation in both mice and humans (**Figure 6D**).

### 4.2 RSS modulation of B cell-mediated humoral immunity

#### 4.2.1 Direct antibody persulfidation and functional consequences

H_2_S regulation of humoral immunity operates at multiple levels: beyond cellular effects on B cells, H_2_S directly modifies antibody molecules, the key effectors of humoral immunity. Antibody function highly depends on abundant disulfide bonds, maintaining correct assembly of heavy and light chains and conformational stability of antigen-binding regions (167, 168). Studies found that pharmacological concentrations of H_2_S treatment (which rapidly generates a broad RSS pool, including persulfides/polysulfides) cause disruption of disulfide bonds between antibody heavy and light chains, accompanied by antibody persulfidation modification (169).

Disulfide bond disruption-induced antibody conformational changes produce multifaceted functional impacts. First, H_2_S-treated antibodies exhibit significantly reduced antigen-binding capacity. Second, at the functional level, H_2_S effectively blocks antibody-mediated red blood cell agglutination reactions and antibody-coated microsphere aggregation. More importantly, H_2_S significantly inhibits antibody-dependent multiple effector functions: in glomerular mesangial cells, it substantially suppresses antibody-induced complement-dependent cytolysis (CDCS); in Jurkat cells, it blocks anti-CD95 IgM antibody-triggered cell apoptosis. Furthermore, H_2_S significantly inhibits the alternative activation pathway of the complement system (169). These findings reveal that pharmacological concentrations of H_2_S can inhibit humoral immune responses by directly sulfhydrating effector molecules.

#### 4.2.2 Indirect B cell regulation: evidence and knowledge gaps

Beyond direct antibody effects, RSS may indirectly influence B cell-related responses by modulating the immune microenvironment. In benign prostatic hyperplasia (BPH) rat models, diallyl sulfide (DAS) demonstrates significant immunoregulatory effects. Histological changes and immune-inflammatory cascades accompany testosterone propionate (TP)-induced BPH, while DAS or finasteride (Fin) treatment ameliorates these abnormalities (170).

DAS treatment reduces prostate weight by 60%, decreases serum testosterone and dihydrotestosterone (DHT) by 68% and 75% respectively, while androgen receptor (AR) and prostate-specific antigen (PSA) protein expression decline. DAS significantly alleviates the immune-inflammatory microenvironment, manifested by reduced CD4^+^ T cell protein expression and related inflammatory cytokines, while inhibiting insulin-like growth factor-1 (IGF-1), transforming growth factor-β1 (TGF-β1), and phosphorylated ERK1/2 signaling pathways (170). Although this study primarily focuses on T cells and tissue microenvironments, considering that B cells participate in immunopathological processes by producing pathogenic antibodies, and immune microenvironment changes affect B cell activation, differentiation, and antibody generation, these findings provide important clues for exploring RSS roles in B cell-mediated autoimmune diseases and metabolic diseases.

Current understanding of RSS regulation of B cells remains limited. Key unresolved questions include (1): Direct effects on B cell activation and differentiation. Do RSS modulate B cell receptor (BCR) signaling, germinal center formation, or plasma cell differentiation? (2) Class-switch recombination regulation. Do RSS influence antibody isotype switching (IgM→IgG/IgA/IgE) via redox-sensitive enzymes? (3) B cell-T cell crosstalk. How does RSS dysregulation in T follicular helper (Tfh) cells impact B cell responses? (4) Metabolic programming. Does cysteine/GSH availability (Section 3.3.1) similarly constrain B cell proliferation and antibody secretion?

Addressing these knowledge gaps requires systematic investigation using B-cell-specific RSS metabolic enzyme knockout models, proteome-wide profiling to identify B-cell-specific persulfidation targets, and functional assays integrating BCR signaling, germinal center dynamics, and antibody production.

## 5 Summary and perspectives

### 5.1 Integrative summary: RSS as multilevel immune coordinators

This review systematically dissected RSS immunoregulatory mechanisms across innate and adaptive immunity, establishing RSS as central coordinators integrating redox metabolism with inflammatory programming and antigen-specific responses. Three mechanistic tiers emerge:

Transcriptional control. RSS modulate master transcription factors governing immune cell fate: NF-κB (exhibiting context-dependent duality: suppression in acute inflammation vs. baseline activation for immune surveillance, Section 3.1.1), Nrf2 (via Keap1 Cys151 persulfidation for antioxidant defense, Section 3.1.2), and NFYβ (recruiting Tet1/Tet2 for Treg lineage stability, Section 4.1.2). This transcriptional reprogramming orchestrates macrophage M1/M2 polarization, neutrophil chemotaxis inhibition, and DC tolerogenic cytokine secretion.

Metabolic and organellar homeostasis. RSS maintain cellular bioenergetics through multiple organelles and metabolic circuits: Mitochondria: PINK1/Parkin-mediated mitophagy clears damaged mitochondria in sepsis (Section 3.1.3). Glycolysis-Mitochondria Axis: Regulating the glycolytic switch and HIF-1α stabilization to control bactericidal capacity in tuberculosis (Section 3.1.4). Golgi Apparatus: Mitigating Golgi stress to preserve CD8^+^ T cell effector function (Section 4.1.3). Cysteine-GSH Axis: The System xc^−^-GSH axis serves as a foundational checkpoint for DC differentiation and antigen presentation (Section 3.3.1). Ferroptosis Resistance: Cysteine availability governs glutathione synthesis to protect CD8^+^ T cells from lipid peroxidation (Section 4.1.3).

Effector function modulation. RSS directly targets immune effector mechanisms: inhibiting pathological NETosis through a three-axis mechanism (ROS-MAPK suppression, platelet-neutrophil crosstalk blockade, and miRNA epigenetic regulation, Section 3.2.3); enhancing T cell activation via autocrine H_2_S-mediated immunological synapse optimization (Section 4.1.1); and disrupting antibody function through direct disulfide bond persulfidation (Section 4.2.1).

Notably, emerging evidence highlights the structural and functional diversity of RSS: while H_2_S often promotes activation (e.g., T cell proliferation), persulfides and polysulfides (e.g., GSSSG, driven by CARS2) act as a distinct brake to limit excessive CD4^+^ T cell proliferation (Section 4.1.4). Furthermore, RSS exhibit profound duality in cancer: exogenous donors enhance anti-tumor immunity by targeting MDSCs, whereas endogenous CBS/CSE overexpression in tumor cells drives immunosuppression (Section 3.3.3).

### 5.2 Critical knowledge gaps and mechanistic challenges

Despite substantial progress, several fundamental questions remain unresolved:

Context-dependent molecular switches. The determinants governing RSS bidirectional regulation remain incompletely defined. How does H_2_S switch between NF-κB suppression (LPS-induced acute inflammation) and activation (baseline immune surveillance)? What dictates H_2_S’s opposite effects on neutrophil infiltration (inhibition in lung injury *vs*. promotion in abdominal aortic aneurysm, Section 3.2.2)? Resolving these paradoxes requires systematic analysis of how RSS concentration kinetics, coexisting signaling molecules, and the cellular metabolic state synergistically determine functional outputs.

Species specificity: A critical emerging gap is distinguishing the roles of H_2_S from persulfides/polysulfides. While H_2_S donors enhance T cell activation (Section 4.1.1), CARS2-derived polysulfides inhibit CD4^+^ T cell proliferation (Section 4.1.4). Furthermore, CSE regulates DC IL-12 production via cysteine pool modulation independent of H_2_S (Section 3.3.1). Future research must disentangle these overlapping yet distinct metabolic and signaling pathways.

Dose-response relationships and hormesis. H_2_S effects follow hormetic curves—low-to-moderate concentrations may promote immunosuppression (e.g., tumor Treg activation via ENO1 persulfidation), while high-concentration pulsatile exposure produces anti-tumor effects (MDSC inhibition). However, optimal therapeutic windows across different immune subsets lack systematic characterization.

Immune cell subset heterogeneity. Why do H_2_S donors selectively inhibit pathological CX3CR1^+^ moDCs in NASH without affecting normal macrophages (Section 3.3.2)? Do different B cell differentiation stages exhibit differential sensitivity to disulfide bond disruption? Understanding these cell-specific responses is crucial for precision targeting.

### 5.3 Translational roadmap and clinical prospects

Next-generation RSS regulatory tools. To overcome the poor spatiotemporal control of traditional donors, innovation is required in three tiers: (1) Targeted delivery: Nanocarriers conjugated with cell markers (e.g., CD206, CD8) or disease-specific homing peptides. (2) Smart responsive platforms: Hydrogels triggering release in response to pH (e.g., JK-1 donors), ROS, or enzymes, as seen in recent diabetic wound management studies (Section 3.1.5). (3) Metabolic engineering: CRISPR-based editing of RSS pathways (CSE, CBS, CARS2) to modulate endogenous capacity.

Biomarker development. Clinical translation requires non-invasive quantification. Promising candidates include sputum H_2_S for airway inflammation (Section 3.2.1), circulating persulfidomics signatures, and genetic polymorphisms in sulfur-metabolizing enzymes for patient stratification.

Disease-specific strategies. Metabolic Diseases: Topical slow-release hydrogels for diabetic wounds (targeting NETosis/macrophages) and systemic CSE activators for vascular protection. Autoimmune Diseases: Slow-release donors (e.g., GYY4137) to enhance tolerogenic DC/Treg axes (Section 3.3.2). Tumor Immunotherapy: A dual strategy combining tumor cell CBS/CSE inhibition (to disrupt endogenous immunosuppression) with exogenous donors (to target MDSCs and protect CD8^+^ T cells from ferroptosis). IBD: Targeting the CARS2- cysteine persulfide metabolism axis to control T cell expansion (Section 4.1.4).

### 5.4 Concluding remarks

RSS have emerged as sophisticated immune coordinators operating through transcriptional reprogramming, metabolic homeostasis, and direct effector modulation. The evolution from simple donors to smart responsive biomaterials demonstrates translational progress. Moving forward, distinguishing H_2_S from reactive persulfides/polysulfides and decoding context-dependent mechanisms will be critical. As this field matures, RSS promise transformative impacts across metabolic diseases, autoimmunity, and cancer immunotherapy.
